# PhMYB37 Promotes Shoot Branching in Petunia

**DOI:** 10.3390/genes13112064

**Published:** 2022-11-08

**Authors:** Lili Dong, Tianyin Yang, Di Gao, Tian Wang, Xinyi Deng

**Affiliations:** College of Horticulture, Anhui Agricultural University, Hefei 230036, China

**Keywords:** petunia, shoot branching, *MYB37*, subcellular localization, functional analysis

## Abstract

Petunia is one of the world’s most important flowers, and its branch development has long been a source of discussion. MYB transcription factors have been identified as important plant branching regulators. In this study, 113 R2R3-MYB genes were identified from the petunia genome. *PhMYB* genes, closely related to *RAXs*, were expressed at greater levels in axillary buds and roots. Decapitation and 6-BA did not regulate the expression of *PhMYB37*. PhMYB37 was localized in the nucleus. Heterologous overexpression of *PhMYB37* promoted shoot branching in transgenic Arabidopsis while silencing of *PhMYB37* inhibited shoot branching. These results suggest that *PhMYB37* plays a critical and positive role in petunia shoot branching.

## 1. Introduction

Shoot architecture impacts the decorative qualities of plants in addition to helping them adapt to their environment, secure their survival, and promote reproduction. Most leaves have axillary meristems to generate axillary buds. These buds have the potential to sprout right away, develop into lateral branches, or go dormant. For many years, much research has been carried out to determine the processes behind axillary meristem initiation and bud expansion. The generally recognized view is that hormones, genes, and environmental variables all play significant roles in controlling shoot branching [1].

MYBs are one of the most prevalent family transcription factors in plants and they play a role in several processes that occur within plant cells, including secondary metabolism, hormone signaling, environmental stress, cell development, and organ growth [2]. The MYB transcription factors of most plants are characterized by an MYB domain containing 50–53 amino acids that encode three putative α-helices. The second and third α-helices form a helix-turn-helix (HTH) structure and bind to the DNA major groove [3]. The MYB proteins are classified into four groups (4R-MYB, 3R-MYB, 1R-MYB/MYB-related, and R2R3-MYB) according to the number of the repeat (s) in the MYB domain [4].

Studies have shown that MYB transcription factors can regulate shoot branching in plants. In tomato, *Trifoliate* and *Blind* regulate the development of lateral organs and the formation of axillary meristems [5,6]. Overexpression of *GmMYB181* caused a significant increase in Arabidopsis branch number [7]. *REGULATOR OF AXILLARY MERISTEM1* (*RAX1*), *RAX2*, and *RAX3* encode members of the MYB-like transcription factors *MYB37*, *MYB38,* and *MYB84*, respectively. *RAX1* is expressed in the central domain within the boundary zone separating the shoot apical meristem and the leaf primordial [8] and is considered a marker gene for axillary meristem initiation. *RAX3* is expressed in the axillary meristem of the central tissue, which can establish and maintain the growth environment of stem cells and promote the formation of the axillary meristem (AM) in the early stage [9]. The *RAX* genes have redundant functions in promoting AM initiation, and the knockout triple mutant *rax1rax2rax3* significantly reduced branching [5]. Although some *MYB* genes that regulate shoot branching have been identified, more *MYB* genes with this function need to be discovered.

Petunia branching regulation is a topical area of study. Due to this, petunia’s genome sequencing was recently completed, enabling genome-wide identification of specific gene families [10]. In this study, we will identify petunia’s whole genome *MYB* genes and analyze the expression characteristics and subcellular localization of PhMYB37. The number of branches under different expression levels of *PhMYB37* will be counted by transgene and virus silencing to clarify the function of *PhMYB37*. This study will provide a theoretical basis for further revealing the MYB family genes’ function in regulating petunia’s growth and development.

## 2. Materials and Methods

### 2.1. Plant Growth

With a photoperiod of 16 h/8 h (light/dark), a temperature of 24 °C, and a light intensity of 3500 LX, Petunia×hybrida cv. “Mitchell Diploid” seedlings were planted in pots (10 × 10 cm) and placed in the tissue culture chamber.

### 2.2. Identification of R2R3-MYB Proteins

Based on the published amino acid sequences of petunia MYB transcription factors [11], amino acid sequences related to petunia MYB transcription factors were searched in the petunia genome database (https://solgenomics.net/organism/Petunia_inflata/genome, accessed on 1 February 2021) using BLAST. The obtained sequences were tested for their conserved MYB domains using the online search tool Pfam (http://pfam.janelia.org/, accessed on 3 February 2021). The petunia MYB members containing two repeats were characterized as R2R3-type MYB proteins.

### 2.3. Expression Analysis of Petunia R2R3-MYB Genes

Thirty-day-old petunia seedlings were employed as experimental material. For RNA extraction in the tissue expression experiment, roots, stems, leaves, buds, and flowers were collected. One portion of RNA was utilized to detect gene expression levels with quantitative real-time PCR (qRT-PCR), while the remaining portion was packaged in dry-ice-filled foam crates and delivered to Beijing Novogene Technology Co., Ltd. (Beijing, China) for transcriptome sequencing. Subsequently, Log2, predicated on the Fragments Per kb per Million reads (FPKM) value, was utilized to generate the heat map using the ClustVis application (https://biit.cs.utee/clustvis/, accessed on 3 February 2021).

Petunia seedlings were separated into three groups for the screening experiment. The first group served as a control, the second group was decapitated, and the third group was treated with 6-BA (50 μM) to the fourth buds from top to bottom. After six hours, the treated buds were harvested for RNA extraction. The expression level of *PhMYB37* was detected by qRT-PCR with primers MYB37-qRT-F/MYB37-qRT-R to analyze the expression characteristics of different tissues and different treatments, with *PhGAPDH* employed as the internal control (Table S2). Each treatment contained three biological replicates, and at least 20 plants were collected and pooled per replicate.

### 2.4. Subcellular Localization

The coding areas of *PhMYB37* were amplified by polymerase chain reaction (PCR) with primers MYB37-SL-F/MYB37-SL-R (Table S2) and then fused to the pSuper1300-eGFP plant expression vector by homologous recombination. The fusion plasmid pSuper1300-PhMYB37-eGFP was subsequently transformed into *Agrobacterium tumefaciens* GV3101 and positive clones were chosen for temporary infiltration into *Nicotiana benthamiana* leaves for the subcellular localization experiment. The GFP fluorescence was observed using a confocal microscope after 48 h of cultivation.

### 2.5. Arabidopsis Transformation and Phenotype Analysis

The constructed pSuper1300-PhMYB37-eGFP was delivered into Arabidopsis using the *A. tumefaciens*-mediated flower-dipping technique [12]. The infiltrated plants’ seeds were harvested and were then placed in a Murashige and Skoog (MS) medium containing 50 mg/L kanamycin. After 15 days of germination, kanamycin-resistant seedlings were transplanted into soil and cultured in a growth chamber. For the purpose of phenotypic analysis, the height of the main stems and the number of rosette branches (bud length ≥ 10 mm) were assessed.

### 2.6. Construction and Transformation of Virus-Induced Gene Silencing (VIGS) Vector

In this investigation, the TRV-based vectors pTRV1 (pYL192) and pTRV2 (pYL156) were utilized [13]. A 300 bp fragment of *PhMYB37* was amplified using the primers MYB37-vigs-F/MYB37-vigs-R (Table S1). Following BamHI and EcoRI restriction enzyme digestion, the PCR products were cloned into pTRV2 to create pTRV2-PhMYB37. By using a freeze–thaw technique, the target gene fragment-carrying recombinant plasmid and an empty vector as a control (pTRV2-0) were transformed into the *A. tumefaciens* strain GV3101. After shaking cultivation to an optical density at 600 nm (OD600) of 0.8–1.0, one positive clone of each construct was chosen. Then, cells were collected and resuspended in medium containing 10 mM 4-morpHolineethanesulfonic acid, 10 mM MgCl_2_, and 200 mM acetosyringone. pTRV1 cultures were combined in a 1:1 ratio with pTRV2-PhMYB37 or TRV2-0 cultures. Agrobacterium cultures were injected using syringes into the leaves of petunia seedlings that were randomly chosen at the four- to six-leaf stage for leaf agroinfiltration. Following infiltration, seedlings were grown in a cultivation space. The number of branches (bud length ≥ 10 mm) was counted 30 days later.

### 2.7. Statistical Analysis

Three replicates were used to perform each experiment in this investigation. Data are shown as mean value and standard deviation.

## 3. Results

### 3.1. Identification and Classification of R2R3-MYB Genes

**Figure 1:**
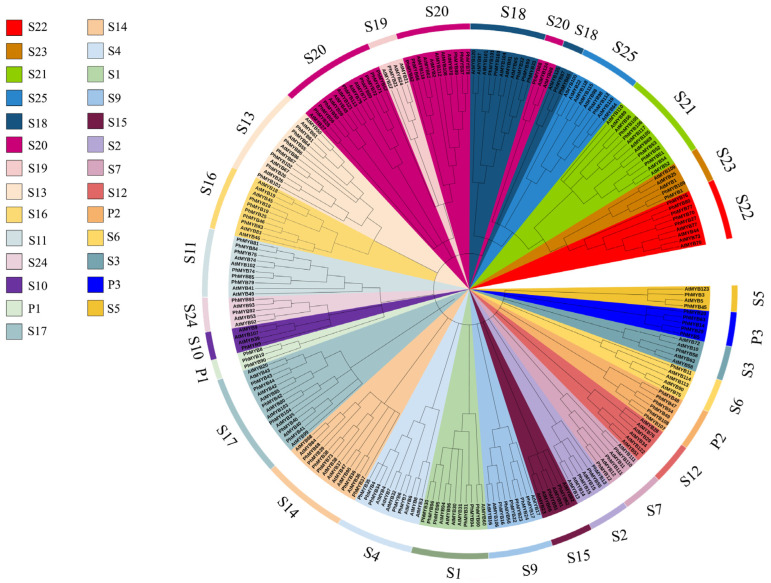
Phylogenetic tree of 113 R2R3-MYB members in petunia and 126 R2R3-MYB members in Arabidopsis. Petunia R2R3-MYB proteins were assigned to 28 distinct subgroups (S1–S25, P1–P3) based on the classification of Arabidopsis. The 28 subgroups were highlighted with different colors.

BLAST was performed using the conserved sequences of the petunia MYB genes. Initially, 151 sequences were obtained from the *Petunia. inflata* genome. After removing the redundant forms or sequences not containing the MYB conserved domains, 145 genes were identified. A total of 26 of these genes were identified as MYB-related, 113 as R2R3-MYB, five as 3R-MYB class members, and one as a 4R-MYB class member. Among the R2R3-MYB members, a total of 28 groups were detected, which were named S1–S25, based on the identified group in Arabidopsis. In addition, we found three new subfamilies, named P1–P3. Phylogenetic tree analysis showed that PhMYB36, PhMYB37, PhMYB38, PhMYB39, PhMYB68, and PhMYB73 were clustered into one branch, and these genes are closely related to Arabidopsis RAX1 (MYB37) and RAX2 (MYB38) (Figure 1).

### 3.2. Expression Analysis of R2R3-MYB Genes in Five Tissues

**Figure 2:**
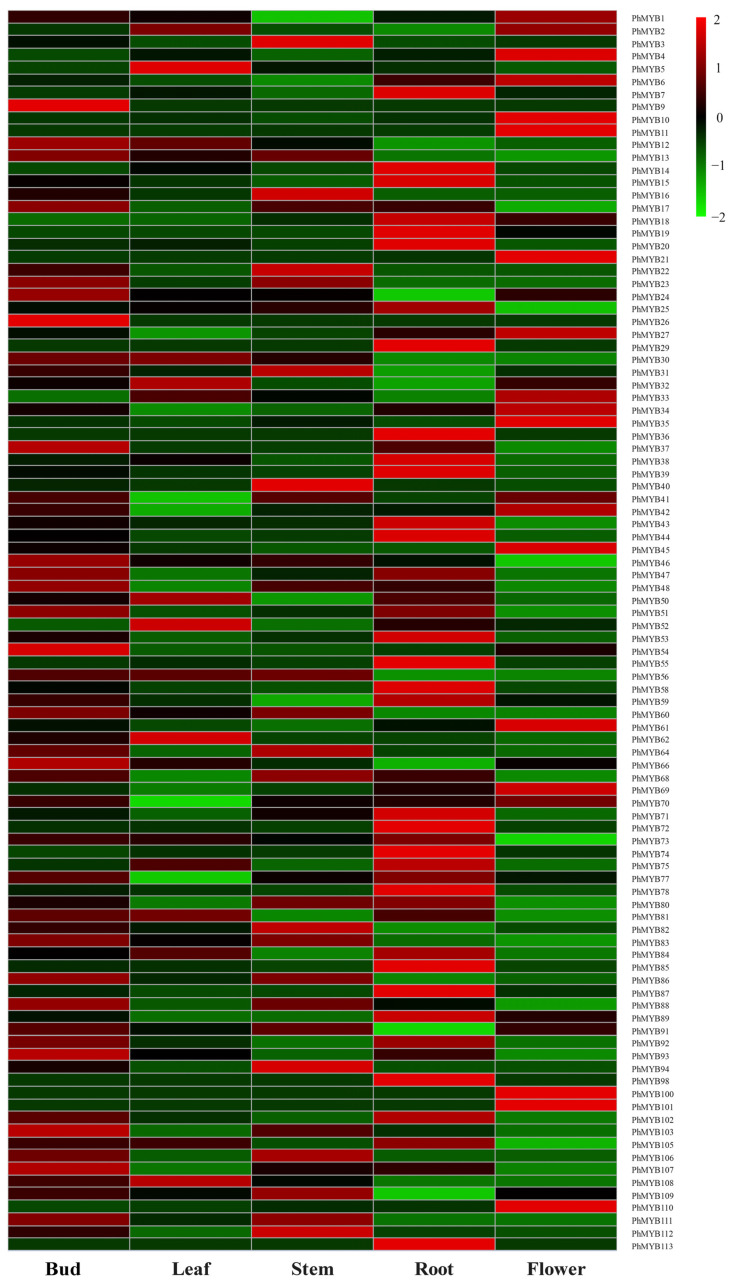
Petunia R2R3-MYB gene expression in different tissues. The R2R3-MYB gene expression levels in the roots, stems, leaves, flowers, and buds is depicted by a heat map. The tissue with the greatest gene expression level was judged to have a gene expression level of two. Blocks with colors represent changes in transcript accumulation relative to the corresponding control, either decreasing (green) or increasing (red).

The expression profiles of *PhMYBs* in five tissues were analyzed using the microarray data to investigate the potential functions of *PhMYB* genes in plant growth and development (Figure 2). The expression patterns of the *PhMYB* genes differed substantially among the five tissues. Fifteen *PhMYB* genes were not detected in any of the five tissues. Twelve, one, six, and five *PhMYB* genes were expressed highest in roots, leaves, flowers, and stems, respectively. In addition, we found that the expression levels of *PhMYB9*, *PhMYB24*, *PhMYB26*, *PhMYB37*, *PhMYB46*, *PhMYB54*, *PhMYB66*, *PhMYB93*, *PhMYB103*, and *PhMYB107* were higher in buds than in other tissues, implying a potential role of these members in shoot branching of petunia. From the phylogenetic tree, we know that *PhMYB37*, expressing highest in axillary buds, was closely related to Arabidopsis *RAX1*, which can regulate the initiation of axillary meristem. Therefore, we chose this gene for further research.

### 3.3. Cloning and Sequence Analysis of PhMYB37

**Figure 3:**
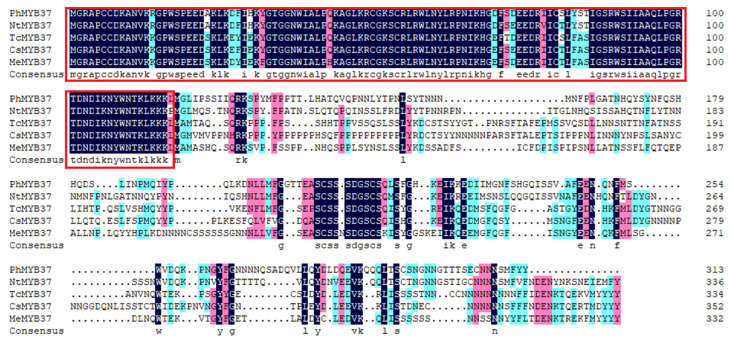
Protein sequence alignment of PhMYB37, NtMYB37, TcMYB37, CsMYB37, and MeMYB37. The accession numbers were listed as follows: NtMYB37, *N. tabacum* (XP_016438630.1); TcMYB37, *T. cacao* (XP_017982221.1); CsMYB37, *C. sinensis* (XP_028089187.1); and MeMYB37, *M. esculenta* (XP_021611134.1). Red frames mark the MYB DNA-binding domain.

*PhMYB37* was isolated using specific primers (Table S1) and the total ORF length of the *PhMYB37* gene was 1565 bp and 313 amino acids were encoded. The homology of the PhMYB37 protein with the other MYB37 protein sequences was compared using DNAMAN software (Figure 3). The results showed that the PhMYB37 was 62.46% similar to NtMYB37 in *Nicotiana tabacum*, 50.58%, 50%, and 48.82% similar to *Theobroma cacao* TcMYB37, *Camellia sinensis* CsMYB37, and *Manihot esculenta* MeMYB37, respectively. The amino acid sequence of PhMYB37 is highly conserved with other MYB37 proteins in the MYB-binding domain.

### 3.4. Expression Analysis of PhMYB37

**Figure 4:**
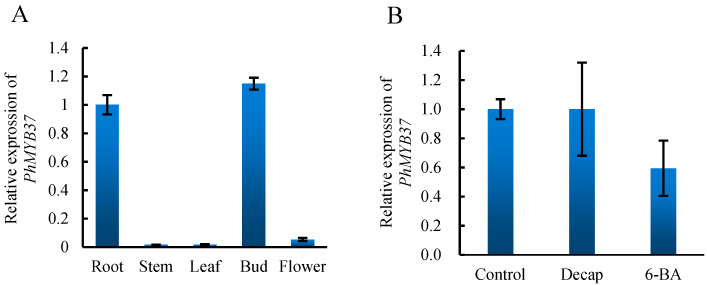
Expression analysis of *PhMYB37*. (**A**) The expression analysis of *PhMYB37* in different tissues. (**B**) The expression analysis of *PhMYB37* under different treatments. Three pools of 20 plantlets served as the source of all samples. Decap represents decapitation.

To confirm the expression patterns of *PhMYB37*, we analyzed the comprehensive expression patterns of *PhMYB37* under different tissues and treatments. As shown in Figure 4, the expression level of *PhMYB37* was the highest in axillary buds, followed by roots, and was lower in stem, flower, and leaf. This result is very similar to that of RNA-seq. Decapitation and cytokinin both regulate axillary bud development [14,15]. Therefore, we examined the expression level of *PhMYB37* under decapitation and 6-BA. We found that the expression level of this gene did not change too much under the two treatments, indicating that these factors may not regulate this gene.

### 3.5. Subcellular Localization Analysis of PhMYB37

**Figure 5:**
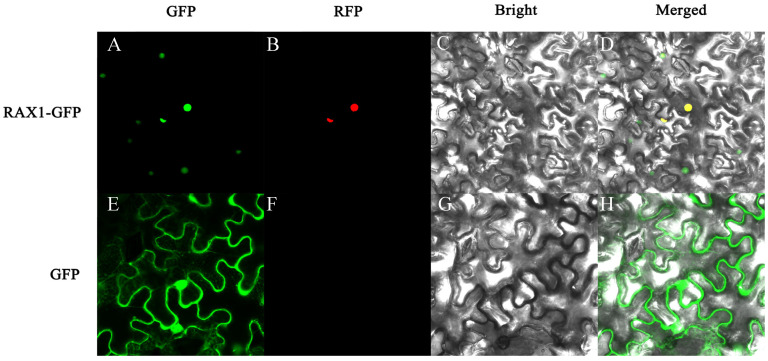
Subcellular localization of PhMYB37 in tobacco leaf epidermal cells. (**A**,**E**) Green fluorescence images of PhMYB37-GFP protein and GFP (control). (**B**,**F**) Red fluorescence image of marker for nucleus localization. (**C**,**G**) Bright-field images of PhMYB37-GFP protein and control. (**D**,**H**) The merged images of PhMYB37-GFP protein and control.

To ascertain PhMYB37’s subcellular location, the *PhMYB37* coding sequence was fused to the green fluorescent protein (GFP) gene activated by the CaMV 35S promoter. A transient expression test in tobacco leaves was carried out. In contrast with the green fluorescence of PhMYB37-GFP and the red fluorescence of the nucleus-localization marker, which were both exclusively seen in the nucleus, the GFP fluorescence of the control was observed across the whole cell (Figure 5). PhMYB37’s subcellular localization pattern was in line with what TFs are known for.

### 3.6. Phenotypes of Transgenic Petunia Plants Overexpressing PhMYB37

**Figure 6:**
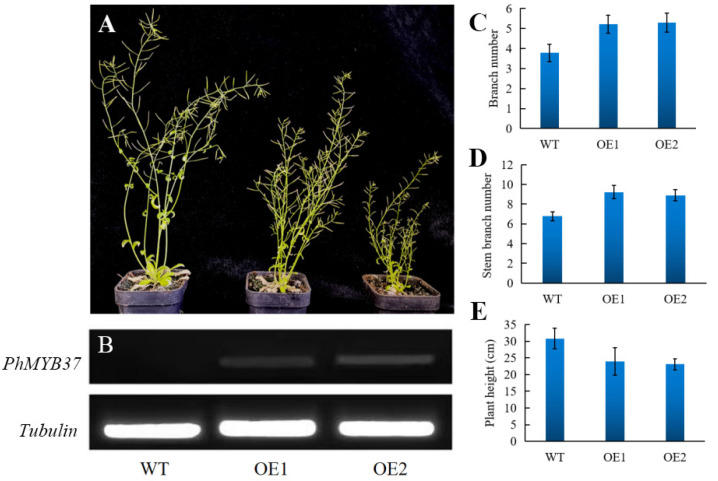
Analysis of the phenotype and gene expression in transgenic PhMYB37-overexpressing Arabidopsis plants. (**A**) Comparisons between the phenotypes of WT and transgenic plants that overexpress *PhMYB37*. Different lines that overexpress *PhMYB37* are represented by OE 1 and OE 2. (**B**) RT-PCR evaluated *PhMYB37* expression levels. *Tubulin* detection served as the control. Twenty plantlets from each of three samples were averaged (±SE). (**C**) Each basal branch’s number is shown (*n* = 15). (**D**) Each stem branch’s number is shown (*n* = 15). (**E**) The primary stem height for each is displayed (*n* = 15).

To ascertain *PhMYB37* function, nine distinct transgenic lines were produced when Arabidopsis was transformed with *PhMYB37*. Two separate transgenic lines (OE 1 and OE 2) from the T_2_ generation were chosen for further phenotypic study. We noticed that *PhMYB37* overexpression resulted in additional branches and a semi-dwarf phenotype (Figure 6A). In contrast with the control, which had 3.8 branches, lines OE 1 and OE 2 had an average of 5.2 and 5.3 basal branches each. In contrast with the control, which had 6.8 branches, lines OE 1 and OE 2 had 9.2 and 8.9 stem branches, respectively.

Additionally, the two PhMYB37-OE lines’ average plant heights were 24.0 cm and 23.1 cm, respectively, whereas control plants’ average height was 30.8 cm (Figure 6D). Consequently, the two PhMYB37-OE lines’ average plant height was reduced by 23.5 percent. According to our findings, *PhMYB37* is an essential regulator of shoot branching and plant height.

### 3.7. Silencing of PhMYB37 in Petunia

**Figure 7:**
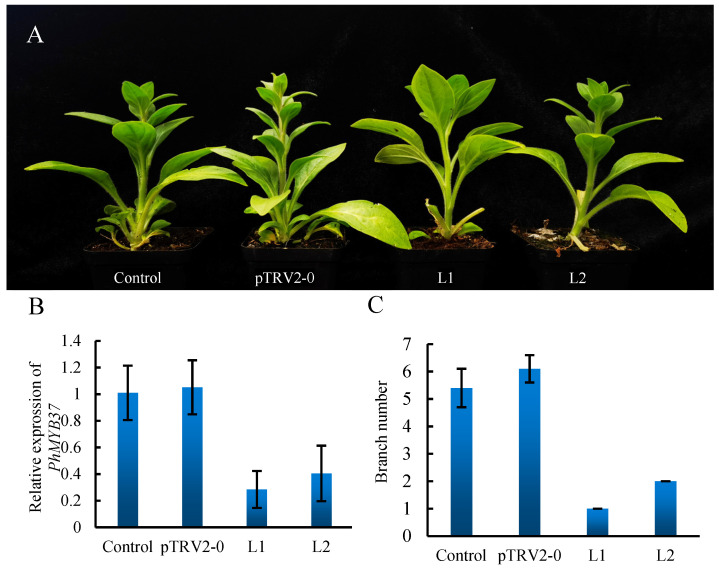
Analysis of the branch number phenotype and *PhMYB37* silencing in petunia. (**A**) Comparison of the phenotypes of control and pTRV2-0 or pTRV2-PhMYB37-infected plants. Different lines that were infected with pTRV2-PhMYB37 are represented by L1 and L2. (**B**) qRT-PCR was used to detect *PhMYB37* transcripts in the total RNA of leaves. The SD of three biological replicates is displayed in error bars. Three pools of 20 plantlets each included four samples: control, pTRV2-0, Line 1 (L1), and L2. (**C**) The branch numbers of the control, pTRV2-0, L1, and L2 plants are displayed (*n* = 15).

*PhMYB37* expression was measured using qRT-PCR to demonstrate *PhMYB37* suppression at the molecular level. *PhMYB37* was likely repressed since the average expression level of the gene was 4.3 times greater in pTRV2-0 plants than that in pTRV2-PhMYB37 seedlings (Figure 7B). With *PhMYB37* silenced, the number of petunia branches dropped from 3.8 to 0.5 (Figure 7C). This demonstrates a relationship between the *PhMYB37* expression level and petunia’s branching pattern.

## 4. Discussion

The MYB family is one of the plants’ most crucial transcription factor families. In recent years, MYB gene family members in various plants have been identified by analyzing the available whole-genome sequences. In the present investigation, we identified 113 R2R3-MYB genes in the petunia genome and performed expression profile analyses in various tissues. As shown in Figure 2, *R2R3-MYB* gene transcript abundance was associated with different tissues. Approximately 39.8% (45 of 113) of the *R2R3-MYB* genes were expressed in all tissues, suggesting the vital roles of *R2R3-MYB* genes in controlling plant growth and developmental processes. We found that *PhMYB55* and *PhMYB59* were significantly expressed in roots compared with other tissues. This was consistent with a previous report that *AtMYB59* regulated root development by fundamentally controlling cell cycle progression [16]. *PhMYB91*, the homologous gene of *AtMYB91*, had higher expression levels in stems and leaves, which is consistent with a previous report that *AtMYB91/AS1* regulated shoot morphogenesis and leaf patterning [17]. *PhMYB9*, *PhMYB17*, *PhMYB37,* and *PhMYB107* showed the highest expression in buds, and these genes are closely related to *RAXs*, suggesting that the four genes may play essential roles in the shoot branching of petunia.

In order to further study the function of MYBs in the development of petunia branches, we selected *PhMYB37* for further study. qRT-PCR was used to analyze the expression characteristics of different tissues and treatments. The results showed that the tissue-specific expression analysis of *PhMYB37* was very similar to the results of RNA-seq, which also confirmed the high quality of our transcriptome sequencing. We found that *PhMYB37* was not regulated by decapitation and cytokinin, which suggested that decapitation and exogenous cytokinin might not regulate the changes of the axillary meristem. We transformed *PhMYB37* into Arabidopsis and obtained overexpression in transgenic plants. The results showed that overexpression of *PhMYB37* caused an increase in the number of branches, both the basal branches and the stem branches. However, compared with the phenotype of plants overexpressing *WRKY71* or other genes, overexpression of *PhMYB37* did not increase the number of branches too much. However, after reducing the expression of *PhMYB37* by viral silencing, the bud outgrowth was significantly suppressed. This indicates that *PhMYB37* is necessary for regulating the development of axillary buds and needs to maintain at a particular expression level to ensure the normal development of axillary buds. However, the increase in the gene expression product cannot strongly promote the formation and germination of more axillary buds.

## 5. Conclusions

In our study, 113 R2R3-MYB genes were identified and classified into 28 groups. Expression analysis indicated that some *R2R3-MYB* genes showed tissue-specific expression characteristics in buds. PhMYB37, as a nuclear-localization protein, did not respond to decapitation and 6-BA treatment. The change in expression level of *PhMYB37* by transgenic and virus-silencing experiments resulted in increases or decreases in the number of branches, indicating the important role of *PhMYB37* in petunia shoot branching. This study could provide genetic resources for breeding petunia with different branching characteristics by genetic engineering, and also provide a theoretical basis for studying the branching development of petunia.

## Data Availability

Not applicable.

## Supplementary Materials

The following supporting information can be downloaded at: https://www.mdpi.com/article/10.3390/genes13112064/s1, Table S1. Protein information of PhMYB members; Table S2. Primer names and sequences were used in this study.
