# Supplementary material for: PhMYB37 Promotes Shoot Branching in Petunia
Source: Genes (Basel). 2022 Nov 8;13(11):2064. doi: 10.3390/genes13112064 (PMC9690887; doi:10.3390/genes13112064)
Supplement: Supplementary file 1 [file genes-13-02064-s001.zip › Table S1. Protein information of PhMYB members.pdf]

Table S1. Protein information of PhMYB members.

PhMYB1
MEIHSLEILETSNPVEGGSYGGDSGGEDEMLENESGSSENVVKKNKRTSGERVKGPWSPEEDAI
LSKLVSNFGARNWSLIARGIPGRSGKSCRLRWCNQLDPSVKRKPFSDEEDRLILQAHAIHGNK
WASIARLLPGRTDNAIKNHWNSTLRRRHNGKLRSDPSNVLEDVSAEKSKASSEETQSCGDVNS
LKAIEGKDVSSQENQEDNHHEDKSEAEVQRSDAANDPPTLVRPVARISAFSVYGSLDVPEAQIQ
TPRLTPVQAPDLGISKLLEGCFTDRLVPHQCGHGCCGNASQENNGSSLLGPEFVDYAEPPSFSS
HELAALATEISNVAWCKSGLENSNIEVICNPTTRATSGTSLLPNRPF
PhMYB2
MRKACCDNKEEMHRGAWSKQEDQKLIDYITKHGAGCWRNLPKAAGLLRCGKSCRLRWMN
YLSPNLKRGNFSEDEEDLIIKLHALLGNRWSLIAGRLPGRTDNEVKNYWNSHLRRKLIKMGIDP
KNHRISHYLHRKRLEYWSENSSRGTDHEVVSDAGSSCAKHQPSSLPDLNSPPSIHSSCA
PhMYB3
MGRRPCCSKEGLNKGAWTPMEDKILIDYIKVHGEGKWRNLPKKAGLKRCGKSCRLRWLNYL
RPDIKRGNITRDEEDLIIRLHKLLGNRWSLIAGRLPGRTDNEIKNYWNTNIGKKLQHQRAAAN
SGHAKSRPPVTTQDIVGSGSSLSSSASPCLVVRTKAMRCTKVFIPTPKNTSHDNSITTNCNNDDD
DKVMAVETTALVASSSSSFTLSSILSEQQPISGSSPVSLSGDQFMENSFNFMFNFDMDDPFLSELL
NAAENTTTIGGDQVGDSFNRNEKERSYFPPSSSQSALFSEETQHNDLELWINGFSS
PhMYB4
MGRSPCCEKAHTNKGAWTKEEDERLIAYIKAHGEGCWRSLPKAAGLLRCGKSCRLRWINYLR
PDLKRGNFTEDEDELIIKLHSLLGNKWSLIAGRLPGRTDNEIKNYWNTHIRRKLLSRGIDPSTHR
IMNEPSTQKVTTISFAAGNEDIKDQKISIKAEFEQIKDDEIISKPIKEQCPDLNLELKISPPYQQHSD
RALQQSTTGSGGASTICFTCSLGLKNNKGCSCSRNRSMNVAGYDFLGLKTNGLDYRTLETRTK
PhMYB5
MGRAPCCAKEGLRKGPWSAKEDLLLTNYIKEHGEGQWRSLPNKAGLLRCGKSCRLRWMNYL
KPGIKRGNLSQDEEDLILRLHSLLGNRWSLIAGRLPGRTDNEIKNYWNTHLIKKLKSAGIEPKV
NKSFSKYCSKKQATTDKPRKKQVKKKKNKKIKDQPLVQDTSEPPQVVFIPKPIRISSGHSRNYSV
DQNVALSTSSSNSADNNNNISNNEGKQAEVSFDPLFDEVVLDGCCELSPEWSLPTDDSMLEKV
YEEYLQLISEECFLQLDDPLAENASHHPMLM
PhMYB6
MGRSPCCEKAHTNKGAWTKEEDQRLINYIRAHGEGLLRCGKSCRLRWINYLRPDLKRGNFTE
EEDDLIIKLHSLLGNKWSVIAGRLPGRTDNEIKNYWNTHIKRKLISHGIDPQTHRPLNAAANPA
ATTTITTNTTKNICMDFRNNVDQKPHINIINKTNATDSSNNETKCSSVTTEETQPLELPKKNTQV
MINLELSIGLPFHAKTDHIYSSNSAESTAPYNFLAAAAAPPPTVAAVPVVAMAELVAETACLCW
QIGFQGGQSCGKCKTTTGFYRFC
PhMYB7
MGRSPCCEKAHTNKGAWTKEEDQRLINYIRTHGEGCWRSLPKAAGLQRCGKSCRLRWINYLR
PDLKRGNFTEEEDELIIKLHSLLGNKWSVIAGRLPGRTDNEIKNYWNTHIKRKLISRGLDPQTH
RPLNSTITTNITTSTKNICLDFRKNTSIAATPLSQNDQKPMSSSISSLEDTKCNSSTTEEVQPLDSPP
AAPPVTGEVMINLELSIGLPIFQAKMTDQSSSISSNSAKEMGGKTVCLCWQVGSQNGQQFC
PhMYB8
MGRYPCCKLDNDLKKGPWTAEEDEKLKEYIQGHGHGNWQLVPKRAGLNRCGKSCRLRWTN
YLRPDIIRGGFSDDEEQMIINLHSVLGNKWSRIAAHLPGRTDNEIKNLWNTHLKKKLLKAGIDP
VTHQPINDPRLLLSLSNLMNPLESVLRLQAEVTEMAKIHLIQNIIQVLTPPLIPNLQENNFPMQQ
LYNLALYENIVTNVTHIDEPSPISNSSDMLLSPSVVENNITGFSNVSNVNSEHSLPSLVEATPENSP
SEQNDIIPNYFGSFSETKDFDAWEKSLDDEASNSFWQDIL

PhMYB9
MGRSAESDDMSGLKKGPWTPEEDQQLLDYIHKNGHGNWRALPKLAGLNRCGKSCRLRWTN
YLRPDIKRGKFSEDEERLIIKLHSLLGNKWSAIATRLPGRTDNEIKNYWNTHLRKKLLQMGIDP
VTHRPRTDHINIFNALIGNIPPQLLAAASSNFMNTDNTSINNLTNILFSDTAQQLVHQIQLLQNS
SSLLVHSLFNNAATSTACPLNMEGLNQNFGLQNLCDQNQLQEYNSILMNYQQFLSNTSDSLTG
FALNSSNSTDISATNGCDQLMVMSGTCSTSSSCGNNAASVTHANDDQMKNISKAALHHDTPE
CPSSSNKMPQGNMISDIYPKQELVQPSSASMSTFEPWGQIMGDEEASDSYWRDIIE
PhMYB10
MGRYPCCKDDEDLKKGPWAPDEDEKLMDYINKHGHTNWQLLPKKAGLNRCGKSCRLRWSN
YLKPDIKRGKFSIEEEEMIINLHSLLGNKWSRIAAHLPGRTDNEIKNFYNTHLRKKLLRLGIDPRT
HKPISDLNYLINLSQSFTSNNNNHLMNNPLASVLRLQAEVTEMAKIQQYLQGLFLPPINTNIPF
YSSNIQENFARYSSHLFPPLEGHVTNVTNTQFLDSWLVDCAKSPNLDNSTSCISNSSQSYHQGEL
NSKVDNFEGLNSSSKTYNDYSNSLPVLAPSSPEAAH
PhMYB11
MSTSNASTSGVRKGAWTEEEDLLLRECIEKYGEGKWHLVPVRAGLNRCRKSCRLRWLNYLRP
HIKRGDFSLDEVELILRLHKLLGNRWSLIAGRLPGRTANDVKNYWNTHLRKKNKRARASKAM
KITENNIIKPRPRTFSRPAMNHVSCWNGKSCNKNTIDKNEGDTEIIKFTDEKQKPEESIDDGLQ
WWANLLANNIEIKDLANGNSPTLLHEEIAPLVNIESNLMQEGESGLSDFSVDIDGIWDLLS
PhMYB12
MGRAPCCEKVGLKRGRWTAEEDEILTKYIHANGEGSWRSLPKNAGLLRCGKSCRLRWINYLK
TDLKRGNITPEEEAIIIKLRATLGNRWSLIAEHLPGRTDNEIKNYWNSHLSRKVESLRIPSDERLP
KAVVELAKKGTQQLNKQRRGRVSRSAMQKNRNNLSSTKGTSTASTLSKPQQPPKEINTISMPST
PNIIEKEVLSSTTTSSGQDASNTHLTSDNYASELDNEMLWHEELDPDFIFTCLWNEEGENLETVE
NNNSHNSNTEILSVNVDMVSPGNSKINETSTLMEDGTSLDWDWQYLSEWNEIWAEQEENMS
NNNISSRQLSNEALVEIDPVQQVDYNHSELVAWLLS
PhMYB13
MGRTPCCEKLGLNRGPWSKKEDDILINYIDKHGHPNWRQLPKLAGLLRCGKSCRLRWTNYLR
PEIKRGNFTPEEEDTIIKLHQVLGNRWSVIAAKLPGRTDNEIKNIWHTRLKKKMNQSQIQEKTP
DIREQPSETSKSEDSTNIQENTEISSPKPNSNNQHEPSSSLRSSSSITSSSEGSCSNTTTTSSHIDESRD
QMNLENLLEVDDNFWSEVLWATPADDSKDNNLDFSLSSVEKNYELDSSLNDNWLWDDLFSR
ANEFLLEFPELV
PhMYB14
MQKLHNKNMGTNSKKGPWSPEEDQKLTSYIMKYRIWNWNQMPKFAGLSRTGKSCRLRWVN
YLRPDLKRGPFSMEEVETVIRMYQSLGNRWSAISKELPGRTDNEIKNFFHTHLKKQFGTKVND
HVQVKRKATCKKSKKAKELEMRTRGETQEKPNQSLDMSSSDITFDENHNWDFANFSETSSED
NYCVNSLGAVPIIDSMVILESNPTTSEDPDFSVQDFRDETSINSSNVDFWLELYMAAENLAG
PhMYB15
MVRAPCCEKMGLKKGPWTPNEDQILISYIRKNGHGNWRALPKQAGLLRCGKSCRLRWTNYL
RPDIKRGNFTKEEEDTIIQSHEMLGNRLPGRTDNEIKNVWHTHLKKRLKNYKPPHNTKRHIKN
DDSKGPTTSESSIKSDFNSDTQKNINSPQHSSSEISTVTAESVVMTQSQAINDHDQMMIKHEEME
SSEYFPEIDESFWTDELSTDNNCNHVVTAVPFSSVREENVDMLTTRMEDDMDFWYNEFIKTDD
LQELPEF
PhMYB16
MGRSPCCDKVGLKKGPWTPEEDQKLLAYIEQHGHGSWRALPIKAASSSITYTAYGFDDLQFVG
LQRCGKSCRLRWTNYLRPDIKRGKFTQQEEQTIIQLHALLGNRWSAIATHLFKRTDNEIKNYW
NTHLKKRLTKMGIDPVTHKPMNDTLLSTNDNVQSKNSANLSHIAQWESARLEAEARLVKQSK
FHSYKLDTPRTSTFFDHYSSQELLTPSSSSHLKNLMPVTSGLQDFNFVERSTGFLLCTSADGNLS
SGGMDSDSSGNASEQCEDNNYWNSILDLVNSSPTESTMF

PhMYB17
MGRAPCCDKKGLKKGPWAPEEDEKLIEYIKNNGHNGWRSLPKLAGLLRCGKSCRLRWTNYL
RPDIKRGPFSEEEEKLIIQLHGILGNRWAAIAAQLPGRTDNEIKNLWNTHLKKRLFSMGIDPHIY
EPSSAPSGLVRRPPASSSTRHMAQWESARLQAEARLSKDSHLSIPSLVGKSQSDYILRIWNSEIGE
AFRNFKGVEKTTSESSSPKCGSTSSITTEMDITLTGSSVEGTNQHKDAEWKNCKLYTGNILRGSD
TSSSNDFEESSESALQLLLDVPSKYDLSFLGQSDAYSMYPAFLSESCLNCSSTEHEVCYV
PhMYB18
MGCKQMDKPKQKHKRGLWSPDEDQKLKDYILRHGHGCWSSVPINAGLQRNGKSCRLRWIN
YLRPGLKRGTFSIEEEETVLTLHGVFGNKTDNEIKNHWHSYLKKRVIKKAENEGDARSGYSSPQI
GNEESSHSSMKLASQNSSLDSFEHIECSLLADTDQSSSLQVDFARENCKSNLPRVLFSEWLSFDQ
LNGQDFKNSSNQESKNNFGYNNSVFQDSCMHGPLMNDINQALNRGMVDDMFQPTLEFEDH
ISADGFEELISGEFNINGDMMYI
PhMYB19
MRCKPVESPKKKHRKGLWSPDEDHKLKNYILEHGHGCWSSVPINAGLQRNGKSCRLRWINYL
RPGLKRGAFSIEEEETIMTLHGMIGNKWSQMAQHLPGRTDNEIKNHWHSYLKKRVAKIAENE
AKSQNMDGLSSSSLKLTSRNSSLDSFAQIEGSLTDTDQSISQIEFLKEPRESNFQKVLFAEWFTLD
QFKSQDFQNSGNSNHSRNNNFGYNKSDFQDGFIHGLFMSEGTYGADINIPGVLNNETVDDMF
QASQFKFEDHMSANGFEEFISDEFNITEDVMYI
PhMYB20
MDEIKKGAWSPEEDQKLKAYIKKYGIWNWSQMPKFAGLSRTGKSCRLRWMNYLSPDVKRGPF
SIDEVEIVIKTYQELGNRWSAIAARLPGRTDNEVKNFFHTHLKKHMSLKNDHALMKKKARSK
RVMKKTRENEKTNTGKAQERPAVEVVPTKIPMIGSSSNKNSPSPNSSSSQCNSIITCQENPTDQD
CYNNIISSSRVDQPVIMNMESPFILESNPETSESSLGDTNCNQLIHQFDQYPHDSCNTISDGSSFF
DSFDQFDMIISLFLTSLLNFNLVMVAGLSRTGKSCRLRWVNYLRPDIKRGPFSMEEVEIVMKMY
QECGNRWSTIAANLPGRTDNEVKNFFHTHLKMHLGVKNDVTVKTKGRSKRVKKTNKNEKKL
ADNAQERSADVASPCSSVVICEENHMMDVMDFSKTYQDCYNVSSLVDQSVINMENTVILESNP
ETSETIDAANCQLHQFDYSHLCSSLFDDHRFNSLDMNSFWSD
PhMYB21
MDKRTCNSQDVEVRKGPWTMEEDLILINYIANHGEGVWNSLARSAGLKRTGKSCRLRWLNY
LRPDVRRGNITPEEQLLIMELHAKWGNRWSKIAKHLPGRTDNEIKNYWRTRIQKHIKQADQN
MNKSSKCEQHNDQQAISTSQASTGPTDTIDSYSPPSYTGDTNNNMGNITFQGNFPTETNENIW
SMEDLWSLQLLNDATN
PhMYB22
MGRSPCCEKLGLKKGPWTPEEDQKLMACIEKHGCGSWRALPAKAGLKRCGKSCRLRWINYL
RPDIKRGKFSLQEEQTIIQLHALLGNRTDNEIKNYWNTHLRKRLTKMGIDPITHKPKTNIFGSSQ
LSHMAQWENARLEAEARLVRESKKQISNNNIHDFGSLANTRLAHHQLFNQRSRINNIVPLQA
KVPPFLDVLKVWQDSNWTKPKITKDTSSSSVLDSVLTSMNNGSFDSPTSTNNLFMAPNNNIPRL
VDNSCLVNAGSFMENPEVEIITKEEVQTDQVQINDISSINYMSITDQSSGFPSFIQGFTHLENIVGS
SEDNLEDNKYSYWNTILNSCTSPAGSPVF
PhMYB23
MGRRPCCEKKGLKKGPWTPDEDDKLIEYINKNGHGSWRSLPKLAGLLRCGKSCRLRWTNYLR
PDIKRGPFSHEEEKLVIQLHGILGNRWAAIAAQLPGRTDNEIKNLWNTHLKKRLLSMGIDPQTH
EPASAPNGLLRRPPASLSARHMAQWESARLEAEARLSRESQLLVPSPVGRSETDYFLRIWNSEIG
DAFRKFKKGAKTACHSSASQASSCTKCGSASGIKTEVELRVAAGSPVTGSNQTEFAEWRNEQP
HTEDILQGSDTSSSDAIEDSSESALQLLLDFPSNNDMSFLGQSDSYSMYPFLTASSLICP
PhMYB24
MGRTPCCDKKGLKKGPWTPEEDEKLVEYIKKHGHTSWRSLPNLAGLDRCGKSCRLRWTNYLR
PDIKRGPFSQEEEKLVIQLHGILGNRWAAIASQLPGRTDNEIKNLWNTHLKKRLLSMGIDPQTH

QPSTAPNGLLRRPPTSSSARHMAQWEKARLEAEARLSREPQLLVPSLVGKSETDYFLRIWNSGIG
EAFRKLKKEEKTTCQSPASQESSSTKYGSDSGITTEIDLGLAGSPAAGSNQNEDTKWKNAQPYT
ENFLQGSDTSSSSGLEDSSESALQLLLDFPSPAAERLCILTPLEV
PhMYB25
MGCKIVEKTKQKHKKGLWSPDEDDKLRNYIINHGHGCWSSVPINAGLQRNGKSCRLRWINYL
RPGLKRGAFSLEEEDTILTLHAMFGNKWSQIAQHLPGRTDNEIKNHWHSYLKKRVAKNAENE
SQTQSGNRESLLFSKKWTPQNSSLDSFEHIEGSLADSDQSGSQVDFPKEHQKSNLPKVLFAEWL
CLDKFHGQDFQNSGTFDLTKNNFGYNNSESQHAFMHGLLMNEGSYGSCMNQEVNNGTVDD
MFQPQLKFEDSMSANGFEDFMSGEFINTDVMYI
PhMYB26
MGHHSCCNKQKVKRGLWSPEEDEKLIKYITTYGHGCWSSVPKLAGLQRCGKSCRLRWINYLR
PDLKRGSFSHQEAALIIELHSILGNRWAQIAKHLPGRTDNEVKNFWNSSIKKKLLSHGTLSDHL
SMFTNFTNPNPSPSFDNFYTLINPNNPVNLLPAINSPLILQVDQMNNVNEDLNANSMAPSLIPF
SFDSIPNDPSWFFNYPHSQHDLDYKQDNSNSIVSSNISLNYTDAAGGILMDSRNDPIKSSTHQD
LLVVPNLLPRLSPPAPLQQNFDSMVVNTSTN
PhMYB27
MAQRKESLDRIKGPWSPEEDELLQRLVDQHGPRNWSLISKSVPGRSGKSCRLRWCNQLSPQVE
HRAFTPEEDETIIRAHAKFGNKWATIARLLSGRTDNAIKNHWNSTLKRKCCSVSEDLSFETPEQ
PPLKRSSSVGPGPGPSSPSGSDLSDSGLSCFPQPPLVYRPVARPGPIFPPPPPVQIPNPQKPDPKPD
PVVQPDPPTSLCLSLPGSENQAVQSPTTAQLPPVAPLPAQQGYEYGGATSATEKQFFTPEFLGVL
QEMIRKEVRNYMSAGFVEKNGMCMMQTDAIRNAVVKRMGISKIE
PhMYB28
MAFQLTMQEEELRKGSWHEEEDERLASIVAILGERRWDALAKASGLRRSGKSCRLRWMNYLR
PNLKHGYITADEEHLIVKLQKQLGNKWSKIAKQLPGRTDNEIKNHWRSHLRKKSLIYEQECCG
SNTSKSGQISSASKSDSINTPNNSTGDSISGKDDCSSADSNETGLSDWIPSWSYEQSQMEHHMYL
CRLNLCFCHPQCFSEDNNISTWGDTSSSIWEQ
PhMYB29
MGRKPCCSKVGMRKGAWTAAEDRLLTNYIELNGEGNWRSLPMKAGLLRCGKSCRLRWVNYL
RPGIKRGNFSPEEDDLIIRLHTLLGGRWSLIAGRLSGRTDNEIKNYWNTYLLKKLKAAGIEPKAK
TKKYSTNTRQNKEMKRRRAKNKTDNLLHQAEKPRGKKEDCEVVEKIQVHRPKAIRLTKGYSSS
TSSSFSSSSSQSSCQDNAPKFNFPLSSEEVDNKKEEKIEDNAIYEKLQLFDKLLNGCDISTECSEQT
SSCCYMLDE
PhMYB30
MGRPPCCDKIGVKKGPWTPEEDIILVSYIQEHGPGNWRAVPTNTGLLRCSKSCRLRWTNYLRP
GIKRGNFSEHEEKMIIHLQALLGNRWAAIASYLPQRTDNDIKNYWNTHLKKKLNKKMEGHD
HENINEVGKSSSSSQSNVPKGQWERRLQTDIHMAKQALCEALSLDTPSNVDSPNNNNNPTPKI
LPVDQQPVQTSTTYASSAENIAKLLENWMKNSPKSSESRSSSKTTQMSSLNNLSIGAVSSSSPSEG
TRNVLDSVFSFNSSNNSDVSQSMSIDEGGNFTPENNNATIFQVESKPINLPNFKEDNGIFQQVSK
PNLETQQVPLTLLEKWLLDDANAQAAQEEFMGIGMGMTLGETADLF
PhMYB31
MGRPPCCDKIGVKKGPWTPEEDIMLVSYVQEHGPGNWRAVPNSTGLRRCSKSCRLRWTNYLR
PGIKRGSFTDQEEKMIIQLQALLGNKWAAIASYLPERTDNDIKNYWNTHLKKKLKKLEAASCG
SDLYSKDSCLSSLSSTSKGQWERTLQADINTAKLALQNALSLEKPSAIQEYMATDVKPKNICYPY
IKQEGNNSTSTYASSAENIAKLLKQWTKSSSSTNNSENSKGSSSTQLSCNYNNATNDFESISNFES
FEQSKSDQFSQATTPEESKFYGESKRELDDQVPLSVMLENWLFDENEDLF
PhMYB32
MSITSETDDWMTSKVDMDSPDEANGGGNVGGSLPLKKGPWTSAEDAILVEYVMKHGEGNW
NAVQKHSGLARCGKSCRLRWANHLRPDLKKGAFTLEEERLIIELHAKMGNKWARMAAELPG

RTDNEIKNYWNTRIKRLQRAGLPVYPPDICFLASQNKQNEELAGLSSTDAQHPGLVATNNFDIP
PVEFKNLELNQLLCPPAHVEIPASSLLDIPASGFLAQVHSAPYSSTSLLSTVHPSKRIRGSESMFSG
SNGDLFLASCQYQNGGSLIAQPLGFSSYNHNLTYDGQQSFSNVIPGSHAPLNGNSSTSEPTWAL
KVELPSLQNQTANWDSPSSPLHSLESVDTLIQSPLAGHSESVSLSPRNSGLLDAVLYGSQTMNSS
KDNSNQAKEETSADAVGNSCPDLLEKGWETYGDPISPLSRLPASVFTECTPTTGNSLHELQSTSR
MPGENGCNVKQENADQAPSDGKDDMLNQTIFASNCSSPKTQHTKNNLALKNAFGSGFFDDY
GWDCKQVHSVATSSGQACGRDSAWDAMSAM
PhMYB33
MVAIKIRRDKDMSMSSESDDMMTSKVGVDSPSVEEACGGGNTGGGLPLKKGPWTSAEDAILV
DYVTKHGEGNWNAVQKHSGLARCGKSCRLRWANHLRPDLKKGAFTPEEERRIIELHAKMGN
KWARMAAELPGRTDNEIKNYWNTRIKRRQRAGLPVYPPDICFQAISESKQNEDLGTFSTDGQY
SDFLPVNNYEIPAVEFKKLEFNEPLCPPSLLDIPAGSLLDIPARSLLAQGLNSAYYSRSFLSTMHPS
KRIRGSESLFSALNGDSSPLKNEDSFPTCHQYQNDGTLLAQSLGFSSPYNQNPTSDHHPSSLGVP
GSHAPLNGNSSSSEPSWAKKLELPSLQSQMASWGLPPSPLPSLESVDTMIQSPPTEHTESCNLSP
RNSGLLDAVLYESQTMRASKDNLHQENSGDGADDSCPDLHETEWEAYGDPISPLGHSAASEKF
DGKDDTLNPFFSRPDYLLESNCFSPMQS
PhMYB34
MGRSPCCEKAHTNKGAWTKEEDERLVAYIRAHGEGCWRSLPKAAGLLRCGKSCRLRWINYLR
PDLKRGNFTEEEDELIIKLHSLLGNKWSLIAGRLPGRTDNEIKNYWNTHIRRKLLSRGIDPTTHR
PINEQPTISTQKVTTISFAADNNKDDQDQKIINIKSEFETTSKLQDEIQERCPDLNLELRISPPHDH
QQFNDQSIDELGRRNSLCFACSLGIQNSKDCSCNTNGNGCSSNVSMNIASYDFLGLKTNGLLD
YRTLES
PhMYB35
MGRSPCCEKAHTNKGAWTKEEDERLIAYIRTHGEGCWRSLPKAAGLLRCGKSCRLRWINYLRP
DLKRGNFTDEEDELIIKLHSLLGNKWSLIAGRLPGRTDNEIKNYWNTHIRRKLMSRGIDPTTHR
AINESSTTTQKVTTISFGAGNKNKDIEDLKMINVKAESGLSQEDENSSSSQLFQEQCPDLNLELRI
SPPYQQNQPDHQALKQSPTRGHLCFACSLGIQNSNDCNCSNGCSTNMGMNIASYDFLGLKA
NGVLDYRTLETTK
PhMYB36
MNPRGEDFFGFQKHHITHSFYLYKQVFSSNIFQPTINHKFKKIPFGSKLVEGKKILKMGRAPCCD
KASVKKGPWSPEEDARLKAYIEEHGTGNNWIALPQKIGLKRCGKSCRLRWLNYLRPNIKHGGF
TEEEDNIICSLYISIGSRWSIIAAQLPGRTDNDIKNYWNTRLKKKLLGKRKQSQMNRLLLAGGQ
DLKETNGLEENSLLQNLSNSALERLQLHMQLQSLQNPLSFYNNPALWPKLTPLQQKMIQTLQA
TGLSIENQSSLLAQISPSANQVDQLGQKVGINEFANTMSTRFKVNNEVEKSTVNNGISSSDSPID
FSNQKDVLDTNIGQENTGEIQGIQGFTQAEIDDLILINNKGLIADQFDCFKEMDGSSSRDNLAW
WSNDFDTNTTSSNSWGSSSNNIVQQTEGMYQDYALGYNLQ
PhMYB37
MGRAPCCDKANVKRGPWSPEEDAKLKDFIHKYGTGGNWIALPQKAGLKRCGKSCRLRWLN
YLRPNIKHGDFSDEEDRIICSLYSTIGSRWSIIAAQLPGRTDNDIKNYWNTKLKKKLMGLIPSSIIQ
RKSPYMFPPTTLHATQVQPNNLYTPNLSYTNNNMNFPLGATNHQYSYNFQSHHQDSLINPMQ
IYPQLKDNLLMFGGTTEASCSSSDGSCSQLSFGHKEIKKEDIIMGNFSHGQISSVAFEENQNFMS
WVDQKPNGYFGNNNNQSADQVLLQYDLDQEVKQQQLTSCSNGNNGTTTSECNNNSMFYY
PhMYB38
MGRAPCCDKANVKKGPWSPEEDAKLKEYIEKSGTGGNWIALPHKAGLRRCGKSCRLRWLNY
LRPNIKHGEFSDEEDRVICTLYASIGSRWSIIAAQLPGRTDNDIKNYWNTKLKKKLMGLVSTSQK
IRPLHNQHQIITNYNYYPQIPFHQTSSLASSSPYSSTTTIPCYESTIPTLASSSFLNTASASCTSGISGS
TSGTVLQVQESYVGGPTTSSDGSYTNQMSQGRDQLEYDYGVSNGGENLDFHNYLYNNGIIGLE
DQSTSSKFLNIEAADEKPIINANSCYVQQEQNPLDYSLEEIKQLISTNNVTCNSNFFLDENKIEEK

VMMYY
PhMYB39
MGRAPCCDKNSVKKGPWSPEEDAKLKAYIEQHGTCGNWIALPQKIGLKRCGKSCRLRWLNYL
RPNIKHGGFSEEEDNIILSLYISIGSRTDNDIKNYWNTKLKKKLFGKQRKNLKGNNQKQGSRKG
REMSSSMSMVSNENIIANPSWPELPILQPIPYSNEEPRFNDHSSIRKLLMKLGGKFSDDDNDDEK
PMNEPPSNPQYPMDNSLIQPIYDQDCINMMHSPFTNNTHYNMDAKALCWADTDTERRKLGE
RMGSDTPVVPTVNDGCNFINELEHMMYTNPQKLSGLEMLYVDMLNNKHATTLGGSLDLEDM
NNLVFPLPPLDASNTEGHQHGTLLQGGALNELRLQAMSHPPCVWLQQPGKGWLSEAIMIRES
RVEGLSLNGSK
PhMYB40
MGRQPCCDRVGLKRGPWTIEEDHKLVNFLLNNGIQCWRTVPKLAGLQRCGKSCRLRWINYLR
PDLKRGTLSEDEEDQIIQLHARLGNRWSKIASNFPGRTDNEIKNHWNTRIKKRLKLMGIDPLTH
KPLEPIDHKKYKHQPDDHRTELSRTENSNFDNANNDFSVPNDEYNNMLCGNLEVELWNKSC
KTMSSVTCYSSSTSLDADDSINLSTVSAATTESSSNLAPVEDQQDSIQQWMDSLFSCSVNQLEDD
MFFLRKYN
PhMYB41
MGKQPCCDNVGHNRGPWTVEEDHKLTNFILNNGIQNWRHVPKLAGLVRCGKSCRLRWMN
HLRPDLKKGAFTEDEEYMLIKLQSQLGNRWSKIAAHFPGRTDNEIKNHWNTRIKKKLYTEQTR
DIKEGSTKQKYSGQQSLPSLEYLVKDQQQLKEPIEISEMGSNDVNQNSEPVETISIE
PhMYB42
MGRKPCCDKVGVKKGPWTTEEDKKLISFLVTNNIGQCCWRSVPKLAGLRRCGKSCRLRWTNY
LRPDLKRGLLTEAEENLVIDLHSCLGNRWSKIAARLPGRTDNEIKNHWNTHIKKKLLKMGIDP
VTHEPLNKEEKSRDQSTKTDDIDNKQNGHDDQQVHVVLESTNVTAAATSLELENSSLCSSSSFS
PNENSSCITDESQMVLDTFSENNPLLSSMLHVDAPLIDFQWELSASGTTQKQNLDNENFNWLV
DCQDFGIHDFDFDCFNMEMEYFLDSIGDIKNEK
PhMYB43
MGRQPCCDKVGLKKGPWTADEDKKLINFILSNGQCCWRAVPKLAVILDDGIGAGLLRCGKSC
RLRWTNYLRPDLKRGLLSEYEEKMVIDLHAQLGNRWSKIASHLPGRTDNEIKNHWNTHIKKK
LKKMGIDPVTHKPLSTITNDHPNKQQPKDLPVIQQENLQEIIMPPSSVNDVSEMDIETPIEQSAIS
EIKVEEDNNNKNMETTSCKNNINISFDSTTVEVNNNGFCTDEVPLIEPHEILVPNSESTPSTSSSS
LSTSSSSSSSILEDLKFLPSFDEWPSDYYNMEQNNMGLGWENDFSSTLDFLLNDDNDINQFPRD
DESWKFDQLL
PhMYB44
MGRQPCCDKAGVKKGPWSAEEDKKLINFILNNGQCCWRALPRLAGLMRCGKSCRLRWTNY
LRPDLKRGLLSGYEETMVIELHAQLGNRWSKIASHLPGRTDNEIKNHWNTHIKKKLKKMGIDP
VTHKPITSDQPNIEQPTKDQPITSDQPTIEQPTKNQQEKQNIMPPSTVHVVQEMDIDQNKELVE
TPIQSTVTVTKLEEGTSPKNMEPIQVNNGFCTDEVPLIEPHEILVPSESTPSTSSSSSSSPLEDMQFL
PSFDNWQCDFNNMDNIGINWAHDFSSTLDYLLNDDDNDMKNISLQDWSQVLEV
PhMYB45
MRTPSSSSTTSNKVTPCCSKVGLKRGPWTPEEDEILTNYINKEGEGRWRTLPKKAGLLRCGKSC
RLRWMNYLRPSVKRGHIAPDEEDLILRLHRLLGNRWSLIAGRIPGRTDNEIKNYWNTHLSKKLI
SHGIDPRTHKPLKNSNSSSDDITNKLASSSPPSSSKANDLNPILSPTYISSFQMEEPLGKINTHPGE
ITSLDDQYQSNAILAEYGDDLNIAVTIEEDVEMNCCTDDVFSSFLNSLINEDMFACQNQQTNGT
FQDFDPFMASSSTPSSDQYNPS
PhMYB46
MRKPEFSSSGKNGTNSNNNINNANMKLRKGLWSPEEDEKLMHYMLTNGQGCWSDVARNAG
LQRCGKSCRLRWINYLRPDLKRGAFSPQEEELIIHLHSLLGNRWSQIAARMPGRTDNEIKNFW
NSTLKKRLKNSSSCSTPSPNASDSSSEPCKDNLNMGINQGLIMSMQNHNLMSMFMDSTSSSSSS

MALNTIIEPLPMLEQTLINMPNGLSAPPYLTTQPCMTQGRSIVTNGSLFYGNNHGIFGGNLGME
GELYIPPLESVSIHEYQNVENGNLVERSSQNNNNPTNSMTNLTSHFNSSSNNIKVENFGGVGNY
WEGDELKVGEWDLEELMKDVSPFPFLDFQVE
PhMYB47
MVRTPSIDKNGMKRGAWSEEEDNKLRAYVERFGHPNWRKLPRYAGLMRCGKSCRLRWMNN
LRPGLKKGNYSHEEEQLIIKLHNQLGNRWSTIAAKLPGRSDNDIKNHWHAHLKKRTKTNANS
STMEQMSTESSQSGCQAEQSNSKLPELENYCDPIDTSSPPEVSSSDLSKLFSSCSLLNGMDWIED
DQIRSMEQLSNIDSVEPLLDSFSCWTKPIDNFQTEHCFDNVWSEHFDNFWTQPFL
PhMYB48
MVRTPSVDKNGVKRGAWTEEEDNKLRAYVERFGHWNWRQLPKYAGLMRCGKSCRLRWMN
YLRPGLKKGNYSNEEGQLIIKLHNELGNRWSAIAEKLPGRSDNDIKNQWHSHLKKRAKTNSYS
STLMEQMSTESSQSGSQSEQSCKLSELEACDKKEVTSAAIDRLDSVPPVSLEVSSSSLLNGIMDY
WMEEEDRMFRPSMELLSIMVRTPSIDKSGMKRGSWSEEEDDKLRSYVERYGHPNWRQLPRYA
GLMRCGKSCRLRWMNYLRPGLKKGKYSHEEEQLIIKLHNQLGNRWSTIAEKLPGRSDNDIKN
HWHAHLKKRSKSNTNSSAIMMKQLIECSQSESQDDEHSNSKFAEHDSFSPIDSVQPVSSDVSSSS
VLNGMDCWFEEDNHIFSSMEPLPDFFNFSWTKPIYNFQTEPFDHYWTEAMDNFWTEPFF
PhMYB49
MVRTPCCDENGRKKGTWTPEEDRKLAAYVTKYGCWNWRQLPKYAGENFCTIYTNIAPREGL
ARCGKSCRLRWMNYLRPNVKRGNYTKEEDEIILKLHAELGNRWSAIAAQLPGRSDNEIKNHW
HTSLKKRANYGPNSSESSKKCNKNSESKRKRVENQNASHETILESSHMSPKQSIGEELCSNTTD
YKQQDVASVIREEELYYEEALAEISGNFWTEPFLLDSFSNRFDFRAPSIDCGLVCPPSPFIGHELLS
SFDFDDSNW
PhMYB50
MVQEEIIRRGPWTEQEDFQLVFYVNLFGDRRWDFLAKVSGLKRTGKSCRLRWVNYLNPGLKR
GKMTPQEERLILELHSKYGNRWSKIARKLPGRTDNEIKNYWRTHMRKQAQDQRKNAFISPSSS
FSNCSSNSSANSPAVDSIPITKQNKRNLETAEEKKIYNHEEAEENQNMVYTMDEIWKNVESSQE
TETMSKLPVMASPIWDYCPDSLWMTDFNYFDNQDIPFFTG
PhMYB51
MCSRGHWRPHEDQKLRELVEKYGPHNWNAIAEKLQGRSGKSCRLRWFNQLDPRINRNPFTEE
EEERLLSAHRIHGNRWAIIARLFPGRTDNAVKNHWHVIMARKCRERSKIYAKRATAASATTIAH
QKSTSDEQELSSLMQQDKRQISNEHTTRSFNPFVDAHQQYFFSERFAYPCNLTYNYSLYPKLLHK
DYLFHHAKVNQDEKQRTEFYDFLQVNTDSNKSEVIDHINASARIMRDDEEVEQEAVDLHRSKS
SRGFIDFLSVGDSI
PhMYB52
MCSRGHWRPHEDEKLRELVAKYGPHNWNAIAENLQGRSGKSCRLRWYNQLDPRINRSPFTEE
EEERLLASHRIHGNRWAIIARLFPGRTDNAVKNHWHVIMSRRCRERSKIYSMRNINNSAQNSTT
SPQDTSQNTRRGSPNMNSIDQQLLGRFNYYPNLTFNNSLYPKELYFDHLRHHLKINEDKNQEV
ECYDFLRVNTGSNKSEVIDHIARKGTDDEEVEQEAGYHYPAQSKAPVQFIDFLSVGDSS
PhMYB53
MAEGGGSGDDTRSSCPRGHWRPAEDERLRQLVEQYGPQNWNSIAEKLQGRSGKSCRLRWFN
QLDPRINRRPFTEDEEERLLAAHRVHGNKWALISRLFPGRTDNAVKNHWHVLMARKQREQSK
ICGKRSYPQDNFLSDSKSPSYGFRRRNNNNNNTNIPKTQEGYNYGTKFSLFEFQNPTKDRVFSV
STYSSSPSWNSPDQLFRRNGSHLLKESSNFSGQKSLCQNNLSFSTHGGGGATLFPVPNNYKKTT
ARNPFSYSNNGSDGMTEKVMNISNSTFSFNKILRASIEQQRRQQQQHAEEAIEKKDIPFIDFLG
VGISS
PhMYB54
MVRTPCRDEYGRKKGTWTPEEDRKLAAYVTKNGCLNWRQLPKHAGLARCGKSCRLRWMNY
LRPNVKRGNYTNEEDGIILKLHTQLGNRWSAIAAHLPGRSDNDIKNHWHTSLKKRANYESSK

KYNKNRESTNTKNQASSRRKSTVENENASSLSCSMSTHETILEGSQWSLKQSSSEEISSCTADYQ
QLDIASDIREVKVFEEEAYVAESCGSFWTEPFVVDSFSSRLHDCVAPSIDFELVSPPSPFIGHGFISSF
NLDDYLMLSSLFD
PhMYB55
MVRAPCCEKMGLKRGPWTSEEDQILISFIQRYGHENWRALPRQAGLLRCGKSCRLRWINYLRP
DIKRGNFSEEEEETIIEMHQVLGNRWSAIASRLPGRTDNEIKNFWHTHLKKKLEHNDLRATTTA
KRSPHEMIPRWKIEHHISSNYQASQNIANQYPTCHDEDLQENNSTSDTQANKEESMQPTNTQI
GHDGDASISNDM
PhMYB56
MGRSPCCDKVGLKKGPWTPEEDQKLLAYIEEHGHGSWRALPAKAGLQRCGKSCRLRWTNYL
RPDIKRGKFTLQEEQTIIQLHALLGNRWSAIATHLPKRTDNEIKNYWNTHLKKRLVKMGIDPVT
HKPKNDALLSHDGQSKNAANLSHMAQWESARLEAEARLVRQSKLRSNSFQNPLASQELFTCP
TPSSPLHKPIVTPTKAPGSPRCLDVLKAWNGVWTKPMNDILRADGSTSATVSVNALGLDLESPT
STLSYFENAQQISNGMIQENSTSLFEFVGNSSGSSEGGIMNEESEEDWKGFGNSSTGHLPEYKDG
INENSMSLNSTLKDLTMPMDTTWTAESLRSNAEDISHGNNFVETFTDLLLSTSGDGDGGLSGN
GTDSDNGGGSGNDPSETCGDNKNYWNSIFNLVNSSPSDSAMF
PhMYB57
MEEVKATQVTYSSGMPVTVKLAEDNIEMRKGPWTLDEDSILIHYISLLGQGRWDSLAQFAGLK
RSGKSCRLRWLNYLRPNLRGGKFTPQEQLLILLLHFRFGNRNDYYLCFRWAKIAEHLPGRTDN
EVKNYWRTKVQKHANQLHCNVNSQEFRDVLRYQWLPSLAEQIRVTPSSSLHEYLMASSSTAH
NKTTEVKENISPFLETGIGSTSITSPNVVSSTNSSEAELPPLMDNSLWSFSSSNWCSSFEDGYYLYSS
QDQCHSWASVAGSELVPANETLVSMGKDLASHGNISSEDTKTSYDEEVRRQCIRP
PhMYB58
MGKGRAPCCDKSKVKKGPWSSEEDLRLITFIQKHGHGNWRSLPKQAGLLRCGKSCRLRWINY
LRPDVKRGNFTPQEEATIVKLHQSFGNKWSKIASYLPGRTDNEIKNVWNTHLKKRLMKKIDG
NQAKDGRSCVSPPFSPSSSTSVVSHTNWSQDISTKANEVEFKDDMLAEKEPKEATKSCSPTTSYG
SNLSNLSQVEISSPKGLDMDNWILSPKVHDIHDDGVIEIPSMESDIDFWDMLDILDPSPTTTTTTS
NSDSKQDNQPVCQGVSDSECQKWLRYLENELGLSQSTGANCYECTDAVTSQDKEAHPFTT
PhMYB59
MVQEEVRRGPWTEQEDLQLVFYVKLFGDRRWDFLAKVSGLKRTGKSCRLRWVNYLNPDLKR
GKMTPQEERLVLELHAKWGNRWSRIARKIPGRTDNEIKNYWRTHMRKKAQEQRKKTCVSPSS
SFSNCSSSSSITHEENERNFYDTGGLELLQSADGQKKVSGHEKGESMKVYSMDEIWKDIELSEEN
DTITSNKLPVMANVSPLWDYCPASLWISDEEESKMFPPFSEHVDDHLQVYSFDKKNRTF
PhMYB60
MGRPPCCDKIGIKKGPWTPEEDIILVSYIQEHGPGNWRSVPTNTGNSVGFTIFYILCSSLLYYFLEY
CYNEAISLFLKGLMRCSKSCRLRWTNYLRPGIKRGNFTSHEEGMIIHLQALLGNKWAAIASYLP
QRTDNDIKNYWNTHLKKKLKKFQATGLDSQSLAPSDSNTYQFSWNFHDSKLQQNQNSSTSSS
STLYASSTENISRLLEGWMRSSPNPSTKNIDDEILHENDQNQEVYKNCESSWLHGGRNNTIGSTS
IPKEYYKSVISEKSACDQSGGAVEKSCTNNAPPYTYLEKWLLDESAGQVEELMELPTIFT
PhMYB61
MGRHSCCYKQKLRKGLWSPEEDEKLINHITKYGHGCWSSVPKLAGLQRCGKSCRLRWINYLR
PDLKRGTFSQEEENLIIELHAVLGNKWSQIAARLPGRTDNEIKNLWNSSIKKKLRQRGIDPNIHK
PLSEVEIDEKELANSKNNEKASEGSSDLNFIEVHENSNLRIATEKPKPSASLVTMDRYPNGMSSA
VPPTHEFFLNRFVTSHESSTATCKPLDLASYLSFQQLNYGSNIGLSMNPNTNPLLFNSNSKNSEM
VSHHQFNSNMPNDILPSISNSILTSPVAAAACSSNIELQRNSSFFDNNAFSWGAADHCGKSEKG
ANNIHSSESDTEDIKWSEYLHTQFLPGNTNIHSHQTSIQHLYSEKSGTQFTTEVSLSTTPWLPNQ
QQQPSIQTADLYNKHFQRLPDAFGQFS
PhMYB62

MTSLSKSSSSSSDDDIGLRRGPWTVEEDTLLVHYISHHGEGRWNILAKRAGLKRTGKSCRLRWL
NYLKPDVKRGNLTPQEQLLILELHSKIGNRWSKIAQYLPGRTDNEIKNYWRTRVQKQARHLKI
DSSISECSNLIPPGDSGLNTFIKAHFPLDGESYDMDTFSPATCSFKDVLYYDQMSGENNAPGDVL
ADSFWSMDEF
PhMYB63
MVLLAAAMKNMQEPYDKNVGLNSNKGAWSPEEDQKLISYIMKYRIWNWNQIPKFADLKRGP
FSMEEVETIIRMYQSLGNRWSAISKELPGRTDNEIKNFFHTHLKKQFGTKVNDHVQVKRKASC
KKSKKAKELEMRTRGETQEKPNQSLDMSSSDITFDENHNWDFTNFSETSSEDNYCTVNSLVNQ
EAVPIIDSMVILESNPTTSENHDLYVQDFMDETGINSSNKLHNKNMGTNSKKGPWSPEEDLKLT
SYIMKYRIWNWNQMPKFAGLSRTGKSCRLRWVNYLRPDLKRGPFSMEEVETVIRMYQSLGNR
WSAISKELPGRTDNEIKNFFHTHLKKQFGTKVNDHVQVKRKATCKKSKKAKELEMRTRGETQ
EKPNQSLDMSSSDITFDENHNWDFANFSETSSEDNYCVNSLGAVPIIDSMVILESNPTTSEDPDFS
VQDFRDETSINSSNVDFWLELYMAAENLA
PhMYB64
MGRHSCCYKQKLRKGLWSPEEDEKLVKHITKYGHGCWSSVPKLAGLQRCGKSCRLRWINYLR
PDLKRGTFSQEEENLIVELHAVLGNKWSQIAARLPGRTDNEIKNLWNSSIKKKLRQKGIDPNTH
KPLSEVENEEKASANSNKNNEKVISEDSNDQLNFIEAHESISKHIGIATEKSSTLSTMTNNMDRY
PLIHETNNIVPPTHEFFTKSPHDLASYFSFQQLNNYSPNSIGLSMTNSNTNNLIFNNSTNSKNSH
DMVSDQFNSCTMATDPEEIKWSEYMQTPYLLSANHHQMSQHQDLYGDAKSEAQFITQGSLNL
SNTTWLHQSQQQQSSLQTADLYNKNFQRLPAAYGQYS
PhMYB65
MSEIIKKGPWKEEEDAVLIKHVKKYGPRDWSSIRSKGLLQRTGKSCRLRWVNKLRPNLKNGVK
FSAEEERTVIELQAQFGNKWARIATYLPGRTDNDVKNFWSSRQKRLAKILRNSASQPSKPQKD
NNKEAPALQKVPSVEEPKLSSPAEERSLPMSQCCSSSYMNNSDTINMVPLPELENSTSLPFEPNL
LQFEFTPNDKNYQYIGTQTSLSFPQIPLQTDFGHPLGSQELPMKLEETDFLDFFGQLSTASDIGN
VQVPLVPLCSGPDKSSEIVVKREMDSPLTQDSFIDDFPMDVFDLIDPLPSPSD
PhMYB66
MSKGIVMNSCSHEDEFELRRGPWTLEEDNLLIHYISSHGEGRWNALAKCAGLKRTGKSCRLR
WLNYLKPDIKRGNLTPQEQLLILELHSKWGNRWSKIAQHLPGRTDNEIKNYWRTRVQKQARQ
LKVDSNSKKFVEAIKSLWMPRLLEKMEQQCSSSISSPASSSSKISIEKQSSSLPSPLINHQEPYKSHD
NTCNYDNEKTNSLEHPRIYTSSSMIQEEGYHVESFNQQPDFSSQEMSISECEMGETNWFTDEMG
GSLWNMDEFWQFRKLGDVDI
PhMYB67
MGHHCCSKQKVKRGLWSPEEDEKLIRYISNHGHNCWSSVPKLTGLQRCGKSCRLRWINYLRP
DVKRGSFSEAEERTIIDVHRILGNRWAQIAKHLPGRTDNEVKNFWNSCIKKKPISQGLDPNTHN
LLSRNSTQNNKNNASCKVKSTSVFTLETMPSSNKEVPMDMIKSSLAAFLPLPHNSKTISTAAYN
DNMNSINVPRTTTNIDFPRSSLMESTSNNYLSSLTPPGYGIINYENYCMWAVSGVERHDFGPSIN
GHEEMRIVEQEGGQVVQLQEKVFQAEEVYKINHDQFNINGQKTYVDDANFSFESVDSALMMP
YGIYTSVKSIDHFAWN
PhMYB68
MGRAPCCDKANVKRGPWSPEEDAKLKAYIELHGTGGNWITLPQKVGLKRCGKSCRLRWLNY
LRPNIKHGEFTEEEDNIICSLYISIGSRWSIIAAQLPGRTDNDIKNYWNTRLKKKLLGKQRKDHR
HHKLEMMKENENYFASQDINAYSWPPQPLLFSSLVAPTNEYQQSGSSGNRHNFQYSTEVSAQF
DPSKFSQSSAFVNPLSGNTCNSLSCYYPISNGVITNSFQEYNNYISVGLDHDVLNVSNLQQVDQ
SVIEMVNSSSTIISTTSPEISTSWEELSPIVFPPSVSNFEIQQEISPYFVFEEPRYLGLLKQ
PhMYB69
MTSECVDRMTSKVGVDSSAVEEASGGGNAGGGVPLKKGPWTKAEDAILVDYITRHGEGNWS
AVRKHSGLARCGKSCRLRWANHLRPDLRKGAFTPEEERLIIELHAKMGNKWARMAAELPGRT

DNEIKNYWNTRIKRQQRAGMPMYPPDICYQAFRENKQNKELGTFSSADSHPDFSPINSFEIPAV
EFKTLELSQQRYPPALLDIAANSSLDIPATSLLAQGLNFSCNTRSFLSTMHPSKRIRGLESWFSGL
NDDFFQACHQFENDGSLFAESLGYSSPHTDNLISVHHPAFSGVFNGSHASLNGNSSSEPKWAK
KLELPSLQTQMASWGSPSPLPSLESYDTLIQLPPSEHIDSGSLSPGNSGLLDAVLYESQAFRASKN
NLHQETCDVVDDSCPDVQATWGTHSGPNSPLGHSAASVFSEYTPIFGGSLHEPRSVASLFGEN
GCKIKQEDVDLAPTDRNNDVSNHQIFAWTESCFAPTVLVPQQNRVLCLTLLPFVSPPSESWAQT
LLDFPPPLLQSSSQPATILRSSD
PhMYB70
MDIITKKGKHIDRVKGPWSPEEDELLTQLVNKHGPRNWSLISKSIPGRSGKSCRLRWCNQLSPQ
VEHRAFTIEEDETIIRAHARFGNKWATIARLLNGRTDNAIKNHWNSTLKRKCSSLSADEGNQL
ADQIFQNDNQPPLKRSVSAGSALPVSGFNFSPGSPSGSDSDSSFHVSSSSQCCVFKPVPRTAGVL
PPGLACPMDTSSSPEAGDPPTLLSLALPGVDSAEHSAESTQVKALLLPQATQIPPPPPPLQAVPIQ
HGEQQDKVFVPFSQELLGVMQEMIKTEVRNYMMGIEQHHPQQQRQQQQQQQYHQQQNH
QLPNGIGLGLCIQQATDGFRNTAANRI
PhMYB71
MLCQLMSSEQMGSWGVIEEGWRKGPWTAEEDKLLIEYVKLHGEGRWNSVARLAGLKRNGKS
CRLRWVNYLRPDLKRGQITPHEERIILELHARWGNRWSTIARSLPGRTDNEIKNYWRTHFKKK
AKNSSANSEKSRARLWKRQQFQQLQQQQQQQQQINDHIDIKKMMSLFDQNENKGQLMPQ
GKQEMAMLYPNTTNQQEQVGLFYSMLNGCASVSLPEPSSNEDTMWDGLWNLDDFYGHFINT
STTYNKATPCLQTMATIPPAFY
PhMYB72
MAEQMGNWGVIEDEWRKGPWTAEEDRLLIEYVNLHGEGRWNSVARLAGLKRNGKSCRLRW
VNYLRPDLKRGQITPYEERIILELHAIWGNRWSTIARSLPGRTDNEIKNYWRTHFKKKVKKSID
NSEKTKARLLKKQQFQQHQQEQLKNQIYMKKVMSLLEGNYENEVPILPQRKQEMDFMYQNT
TEQEQGGGFFYSMLNGYATVPVPEVSSNEDMIMWDHGLWNLEDVNANFNTAAYNKTLPCLQ
PLATPFY
PhMYB73
MGRAPCCDKENVKRGPWSPEEDATLKSYIEKHGIGGNWIALPQKAGLRRCGKSCRLRWLNYL
RPNIKHGEFSDEEDRIICSLYANIGSRWSIIAAQLPGRTDNDIKNYWNTKLKKKLMALDLSSQQI
SPNYQSTPTYHSSISALQSSSHNSSLYSSPSPSLSLKSTAYKYTNSHFYPISTATNFPCNETIISSPSSKF
LNSPSYMYNGVEEKQSNSKFLITNGNDYAEKKPKGKGCYDGENSLECSLEEIKKLISTTSIHDNN
FNNFFSDENKIEEQVMMSTDVDHN
PhMYB74
MGRSPCCDKNGLKKGPWTPEEDHKLIQYIQVHGPGNWRNLPKNAGLQRCGKSCRLRWTNY
LRPDIRRGRFSFEEEETIIQLHSVLGNKTDNEIKNYWNTHIRKRLLRMGIDPVTHSPRLDFLDLSS
LLNSTQLNLSSLLGLQALVNPEILKLANTLFLPHSENQELLLLKLQENQLLNPQHIQNSEGPEM
LLQKLLQSQLVNSPLHNQSSIFQHNTNQIHNQIPEIPNCTQQNVSCSSSQSMQGNMGRYLMNG
QIFQENMMLPPQNYSHTSTPDASENSTFQSLNNSSNQNSQNFSFDTPLSGTEEEKESYCSNFMK
FEIPESLDFDDLL
PhMYB75
MGRTPCCDKNGLKKGPWTAEEDQKLIDYIQKHGSGNWRTLPKNAGLQRCGKSCRLRWSNYL
RPDIKRGKFSFEEEETIIQLHSILGNKWSAIAARLPGRTDNEIKNYWNTNIRKKLLRMGIDPVTH
SPRLDLLDLTSIFNPSLYNPSHMNNMSKLLGVQPLLNPEILRLATSLLSSQRQNQNFLLPSNFQE
NQSCDSQVQNQMTPFVQASQIQIPIQNISICTPLSTPCVPFSSEAQVMQQPNMEQFPSNLSYFSA
QNCQQNEWQLNNGIASKMNEHNFPSQNYVFSTLSTPSSSPASFNSNSTCINSSSTTSEGERESYS
SSMLNFDIPNIFDVNEFL
PhMYB76
MEGTTGDKIKGSWSPEEDNMLIKLVDQHGPRNWSLISTGIPGRSGKSCRLRWCNQLSPTVQHR

PFTPSEDAIILQAHALHGNRWATIARLLPGRTDNAIKNHWNSTLRRKRHGPPLSRSGSSSDQSN
SKRHCTRASQEQSSCGLDCDDLGLDGPNGLDGDELGLYGPETSLTLSLPGGGSIDSPMKEDVPV
KESEPLMNSDNKVKEEKRTVEIEETCLVTIMQRMIAHEVRCYIDKLRAQGGLGIGPGVEFEVPK
NP
PhMYB77
MENFNRCSTSTSSSSESSSSESSLNKAERIKGPWSAEEDKILTKLVERYGARNWSLISKYIKGRSGK
SCRLRWCNQLSPNVQHRPFSHAEDETILAAHAKYGNRWATIARLLPGRTDNAVKNHWNSTL
KRRYQQQKNHNTVIFPDMKNGSGSGSGSGSGSRSGSGSCMDYLNVDESPRGTNVKVVVNNC
NSEYDDPMTELSLAPPGMGGDELPERKTESFSAGFWDVMRDVIAKEVREYVASSFNGASTGFP
PhMYB78
MDHHHKTKVGLGRRGEDYRTSNVMNQENIREEDIMDLRRGPWTVEEDFTLINFIAHHGEGR
WNSLARCAGKSCRLRWLNYLRPDVRRGNITLEEQLLILELHSRWGNRWSKIAQHLPGRTDNEI
KNYWRTRVQKHAKQLKCDVNSKQFKDTMKYLWMPRLVERIQAAATTAATSTTTTNTYIQNLE
NQQSVPNMSHMSQFLPIQLENHKKTNSINHSSMTLTLENSSTTTSSDNSIGRQVSPTSDLTDCYY
NFSINQSSNNQDYTPFNQNYGESLISATGYFPQAVDQQNSQWMDSEYVSDNSWNIEDMWFLQ
QQLNNNL
PhMYB79
MGRSPITSDKSGLKKGPWTPEEDLKLIQYIEVHGPGNWRSLPKNAGLQRCGKSCRLRWTNYLR
PDIKRGRFSFEEEETIIQLHSVLGNKWSAIAARLPGRTDNEIKNYWNTHIRKRLLRMGLDPVTHS
PRLDLLDLSSLFNSTQLNLSSLLGLQALVNPQFLRLATTLLTSHTENNQEMLLQRLQANPTV
PhMYB80
MEGSSSTCSSSSSESSLSAGTPKTPRDGNNNKPERIKGPWSAEEDKILTRFVERYGAKNWSLISKY
IKGRSGKSCRLRWCNQLSPEVEHRPFSPAEDDTILAAHAKYGNRWATIARLLHGRTDNAVKNH
WNSTLKRRQQIRNNVSVVESPIIIIRRNDIIVNVNNWDEVDPMTTLSLAPPGCRRRVCRWSFG
PhMYB81
MGRTPCCEKNGLKKGPWTQEEDQKLIDYIQKHGYGNWRVLPKSAGLQRCGKSCRLRWTNYL
RPDIKRGRFSFEEEETIIQLHSILGNKWSAIAARLPGRTDNEIKNYWNTHIRKKLLRMGIDPVTH
TPRLDLLEFSSILNPSLYNSSQLKVSRLLGGQPLVNPEVLRILANSLLSSQYTVKPSELVDQNLLMS
RNLQGNQLSNPQAQNQLPPLVQDISCGPCSNEAQLMQQANLEQFSSNMSNYSSQNCQLNDW
QNQEMPSHLTEDYNYHLHNYGYYQQDQSIMDPPPSDASAFQSNDSNVSYQSILSTPSSSPTPLN
SNSSYINSTTTEDERESYCSNTFNFDIQNMLDANALL
PhMYB82
MKDKEVKARMKRGFWKPEEDLILKNCVETHGEGNWATISEKSGLMRSGKSCRLRWKNYLRP
NIKRGMMSEDEKDLIIRLHKLLGNRWSLIAGRLPGRTDNEVKNFWNTHLNKRSCKGKKKHN
KAEEANHQRKNDREYPAETARNQEVATKTVLDSWIEEMQDFNCSLLSPLPMNSVAFLQDEPFF
PILDDIVLLEAFTSTGKEVWPDIQPFL
PhMYB83
MRKPEHGSVMKEKGNNNNKAKLRKGLWSPEEDEKLMNYMLTNGQGCWSDIARNAGLQRC
GKSCRLRWINYLRPDLKRGAFSPQEEELIVHLHSILGNRWSQIAARLPGRTDNEIKNFWNSTIK
KRLKNNNNNNNNNNNSSNNNTPSPNTSDSSSDLRVLMGGSIFPMQGHDVNVMAGLCMDNSS
STTSASSLQAIVPNNHFNPFPQLDSTNYDIIGAAGLYNLPPCLGQFGSSSGHDGGVVDYGVVEA
YSSMGLGSDFSVPPLESGNKGTMGENSNIVNFSSNVSAVNDYSLFDKKTNGNNQQLMSDSSDQ
SLKVEDYMVGFGNSHHHHWHGESLRIGEFDWEGLLANVSSLPYLDFQVE
PhMYB84
MGRAPCCDKNGLKKGPWTPEEDQKLIDYIKEHGYGNWRTLPKNAGLQRCGKSCRLRWTNYL
RPDIKRGRFSFEEEETIIQLHSVLGNKWSAIAARLPGRTDNEIKNYWNTHIRKRLLRMGIDPVTH
SPRLDLLDISSILNYPALYNNSSHHQVNFSRLLGHVQPLVNPEVLRLATSLLSSQRQNSNFLMPN
NVQENQICISQVQNQVHPMVQVTSQVQNPIQNIPTCTTLSITPSVPFYDEARLMEQFSSNLVNSS

SQNCQVNEWQSSGMASNLTDQDYFNNPLQNYGYYHELDQSIRDPPLSDASTFQSNDSNNFSF
QSVLSNLSTPSSSPTPLNSNSTTTEDERDNSYCSNMLNFDNIPNIWDSTNEFM
PhMYB85
MGRSPCCDKNGLKKGPWTPEEDLKLIQYIQVHGPGNWRTLPKNAGLQRCGKSCRLRWTNYL
RPDIKRGRFSFDEEETIIQLHSVLGNKWSAIAARLPGRTDNEIKNYWNTHIRKRLLRNGIDPVTH
SPRLDLLDLSSLLNSTQFNLSSLLGLQALVNPEVFRLATTLLASHNNENPELLLQKLQENQLLNT
QLQQNQLLNTQLGNHLQVFQPNSQFQNQIQEIPTFTPSNVPCSSSQPMQLGPVELSYLMNGQ
MLPPQYGYCASNVSDSSNLQSLNNSNNQNSSNFSLDSVLSTPLSSTEDEKESYCSNLMKFEIPAS
LNFDDFM
PhMYB86
MGRHSSFVKEKLRKGLWSPEEDEKLYNYITKFGVGCWSSVPKLAGLQRCGKSCRLRWINYLRP
DLKRGMFSQEEEDMIISLHQVLGNRTDNEIKNFWNSYLKKKLIKQGIDPNTHQPLSENQVRNE
TDCKDKASTSTQIQQPFNLSKRNFNYEASRQLTEASKDQLVSKQVFDPLFLYEFQANVNPSGYN
LRPYDHNQNQIEGNTNFGFFSMPSLTNFEQGHMTETDFSESSSSRMSLLYISEAKESSSNSSNMIS
HHNAAGIQMNEMLENSQALSWDVDNKIDSLFQYPYDGIKNEEDFNNPLSSLSEDLSGENLDV
FHQI
PhMYB87
MDHQHAKVDLRSKEAACSTNNIIQKVEEDDMDFKRGSWTVEEDFTLINHIALHGEGRWNSL
ARSSGLKRTGKSCRLRWLNYLRPDVRRGNITLEEQLLILELHSRWGNRWSKIAQHLPGRTDNEI
KNYWRTRVQKHAKQLKCDVNSKQFKDTLRYLWMPRLVERIQASNSSKNQVIQQTSNNNISN
NIIPSMSFTQENSSTTTSSENSLGTQVISQVSDTSDCCYNYSTINQSDTLCYGESLTSPTGYFHQEG
ALDFRTVDIDDNQQTNQFLDVSDNLWNIEDMWFLQEQLN
PhMYB88
MQNLKKSGNSSGDVAAAKPKERHIVSWSQEEDDILREQIRIHGTDNWTIIASKFKDKTTRQCRR
RWFTYLNSDFKKGGWSPEEDMLLCEAQKIFGNRWTEIAKVVSGRTDNAVKNRFTTLCKKRAK
HEALAKENSNSFINLNNKRVIFPDGLSIDKITEAAAPIKKLRMSGVSDVPQDGSSKGNSFGDCG
TTHPLLRHPFAVLAQNFHNAAGNLASHQQVNNMKESTENATDSKTQGTFLKKDDPKIHALM
QQAELLSSLAMKVNTENTDQSLENAWKILQDFLHQTKEGDMLKFQLPEMNIDLDDYKDLMA
DSRSSNEGSRPSWRQPALSEDSAGSSEYSTGSTLLSHALADKTEESQAEGCAHLQDIESELRNSQ
MSDQGGIHESENGTSCRVSTTPDTLPVCDEEKANNGPATAECEFLNTDFSSPLQVTPLFRSLAET
IPTPKFSESERQFLLKTLGMESITPHPGTNLSQPPSCKRALLHSL
PhMYB89
MDHHHHVKVGSSHGGGANCNKNNQQQHDEDMDLRRGPWTVEEDFALMNYIAHHGEGR
WNSLARCAGLKRTGKSCRLRWLNYLRPDVRRGNITLEEQLLILELHSRWGNRWSKIAQHLPGR
TDNEIKNYWRTRVQKHAKQLKCDVNSKQFKDTMRYLWMPRLVERIQAAAANNTTNSNNPS
KSQMVHQTINSNMSGMSEFVPIHDNVVSTNKPRLMSTENSSTAAASSENSFGTQVSPVNSDLT
DCCYNYPPVNQDYFQVSHHQSTTNNDQLCHGESLTSPTGYFHHGTGLDQFQGMDQQQNMN
SSQWMDGGNNFSDSLWNIDDMWFLQQQLNNNNNNIV
PhMYB90
MGRSPCCTNNNLNKGAWTKEEDEILGNYVKIHGAAKWNILCKKAGLNRCGKSCRLRWTNYL
RPDIKRGNFSEEEDSLIINLHSSLGNKWSKIASQLPGRTDNEIKNHWNTKLRKKLNKLGIEPQT
HKQIPNFNLFNSLQMPPLFSQQDVTQLAKLQLLHFQTLLQTINAITTTFPSNPLMPNINYPMQD
DVNGILNFSMPNFEEDDDNLKKGPWTEDEDEKLIDYIKQNGHTNWQSLARKAGLNRCGKSC
RLRWNNYLRPDIKRGRFSPEEEEIIINLHSDLGNKWSKIAAHLPGRTDNEIKNFYNTHLRKKLLR
LGIDPKTHKPISKLNSLVNLSHHLTSNSNYFLSLASVLSLQAQFSLLAKIQQVIQSPLPTINTINLL
SSIQGNILSNSSPQLESLRNETEMSKFLDSSLANTTTTPCLDNTNL
PhMYB91
MRERQRWRSEEDALLRAYVRQYGPKEWHLVSQRMNTPLNRDAKSCLERWKNYLKPGIKKGSL

TEDEQRLVIQLQAKHGNKWKKIAAEVPGRTAKRLGKWWEVFKEKQQREHKENNKVVDPVD
EGKYDHILETFAEKIVKERSVPGLLMATSNGGYLHADAPAPSPQALLPPWLSNST
PhMYB92
MGRSPCCDETGLKKGPWTPEEDQKLINYIKKHGHGSWRALPKLAGLNRCGKSCRLRWTNYLR
PDIKRGKFSQEEEQTILNLHAILGNKWSAIATHLPGRTDNEIKNFWNTHLKKKLIQMGYDPMT
HRPRTDIFNSLPHLIALANLKELVEQSEVARIKYLQYLLQPSMAANLANSMSTSNTCSVANISD
MEAYNNLLNIGTNHLENSTNNIPPPSTTLQAIQDSITFSHLPELQATPNCNFQNSLNEENMVQT
HVISQGENTPTSPWQLPSSLSPPENDNQPINSYINNIGGSCNPPFFGGAHPSVWPEILLEDSLFQ
DIA
PhMYB93
MGRSPCCDENGLKKGPWTPEEDKKLVDYIDKHGHGSWRALPKLAGLNRCGKSCRLRWTNYL
RPDIKRGKFSEEEEQTILHLHSILGNKWSAIATHLPGRTDNEIKNFWNTHLKKKLIQMGYDPMT
HRPRTDLFASLPNIIALASLLQHHPLEDHAVRLQAEAAQVAKLQYLQWLFQSSSSSSNNYPIATS
LNTTQYSNLEDFGPFNLSNSTKESTPSINLSLLENQALFSNENSGSQLLHNPDTSLPLTDTQEVPF
NFQAHLNNNNNNSNNTSGDNGQDFNFEICSPSSPLHNIIPSPPSSSRLPPLTEISISNNQGDASSTN
SSNAAEGTSSYWPELFFEDHFMHEIA
PhMYB94
MGRPPCCDKVGVKKGPWTPEEDIMLVSYVQEHGPGNWRAVPTKTGLRRCSKSCRLRWTNYL
RPGIKRGNFTDQEEKMIIQLQALLGNKWAAIASYLPERTDNDIKNYWNTHLKKKLKKLQETG
DDLFSRENGYFSSSNSTSKGQWERTLQADINMAKQALHNALTLENLSSCVKQESQVSTYASSTE
NIARLLKGWMGSSTNNSDYSKTSSNNVATADSSSCDGTPSAESEIGLMESFKSLFGFESFESSSSD
QFSQTASPDASKLKGEIKKEANEQMPLSVMLENWLLDENTIQGKDDLSNFSFDETNDLF
PhMYB95
MGRPPCCDKIGVKKGPWTPEEDIILVSYIQEHGPGNWRAVPTNTGLLRCSKSCRLRWTNYLRP
GIKRGNFTEHEEKMIIHLQALLGNRWAAIASYLPQRTDNDIKNYWNTHLQKKLKKLQGHDD
QNNQKGKSQSISKGQWERRLQTDIHTAKQALVDALSLDKKTTNVVSPNINSSANSLPVGQTSS
YASSAENISRLLQNWMKNSPKSSQTTSNSTTSQSSFNNLSMVSSSSPSEVTMSATTPEGLDSLFSF
NSSYNNSDVSQSMLTDEVVAGIFQDGSKQNWQNFKDESGIFQKGSKQNMETQVPLTLLENWL
FDDANAQTQEELMGIGVGMEWP
PhMYB96
MGRPPCCDKIGVKKGPWTPEEDIILVSYIQQHGPGNWRAVPTNTGLLRCSKSCRLRWTNYLRP
GIKRGNFTEHEEKMIIHLQALLGNRWAAIASYLPQRTDNDIKNYWNTHLRKKLKKLQGNDDE
NSSSTSQEGISSSNISKGKWERRLQTDIHMAKKALCEALSLDKPIANPTQTPVQESVQPCTTYASS
AENISRLLQNWMKNSPKSSQLSQSNSESTTTQSSSYNNFSMGQVGSSSSPSEGTISVATPEGFDSL
FSFNSSSVDEGNAAIFQVDSKPNLPNLNAANGFFLEESKPTLESQVPLTLLEKWLFDDAINAPA
QEELMGIGMGMALGEAADLF
PhMYB97
MEGRRPSSSYHHYRYHFHHEDELGEIKKGPWKAEEGEVLLNHVKKYGPRDWSSIRSKGLLQRT
GKSCRLYWVNKLRPNLKNGVKFSAEEERTVIELQAQFGNKWARIATHLHGRTDNDAPKFSSSA
DQEEFLSKSQSCSSSYIDNSNMIYLVPLNPNSTDFEANLLQLDFTANEKKLKIDSHIQLPFTKLQN
DFALPLQTHEFPMPNFIDVFGQQLNGSELDNVQVPFVSTCSGPDRSCFENPSSPDSFIDDFPLDM
FDHIEPLPSQSE
PhMYB98
MEFDHSSNQNLSLQPYNNSLIRGEMDDVFCTVSKDYLQDFHHLDLTFNNPHDNLLIETNGYD
SLFDPILNEGNLSSLDHQDFNLNYEFKPFEQNSASGSTLVMKNFENSMDMHTSNDENLLSLCSE
DMKPLSFVNVQDESSCITADNNYYDNKICRRKKNKKVSSKENILSPCMGKLGKGKKKSKSAKG
QWTTEEDRLLIHLVEKFGVRKWSQIAQMLKGRIGKQCRERWYNHLRPDIKKDLWTEDEDRILI
EAHAEVGNKWTEIAKRLPGRTENSIKNHWNATKRRQFSRRKCRTKWARPSCLLQNYIKSLNFE

KSIRSSHDTTINAPKQEPFGNIPDYDLTSEVPEFAFDDKLFDENINMDALMDQIHDRCLDLEIPY
DELPKLMQGDANKEPDSIDLISEI
PhMYB99
MSEQMDSWGMTKQGWRKGPWTSEEDRLLIEYVNLHGEGRWNSVARVAGLKRNGKSCRLRW
VNYLRPDLKKGQITPHEEKLILELHARYGNRWSTIARNLPGRTDNEIKNYWRTHFKKKTKNSR
DKSEKSKVRLVKRQQWQQQKQQQKLNTQTDSNRVVSLNNKKNYYASILNKLGNKIVSLLDK
NENRILPLVTQKENQEMANLYSNTADQEEDDFLNSILNDYTCVSLLESSFNEDMMWDDFWNN
LDEYQCNVSTTI
PhMYB100
MAPDDRGMKNGGASTGRSNGAGSSRQVLKKGPWTAAEDSILMEYVKKHGEGNWNAVQRN
SGLMRCGKSCRLRWANHLRPNLKKGAFTVEEERIIIELHAKLGNKWARMAAQLPGRTDNEIK
NYWNTRLKRRQRAGLPIYPQELQQQNQHENNNQPHSLLSSSYDPQNSTNYNSPSLSLLDIFNP
STMKPSITQQFPINTPSLCLPSTNNNNIFRNTPKGLSLTLPSSMRNSQFSSLPNNNFTQGLSSNSIQ
VPPFQHNYPNFNINRPFTGISSNPNGLICGMGINTINYPSGQSSMPVTASSSENTGSDFGSSDNA
NNYANTNGLSRGNSGLLEDLLEESQTLNRPGMKIEDNFLDLKEDQEADYKGKSMLWEDYGLV
EDAEEAILTEESAYSFAHGVDHVAQNKNSESSSPHSPPNSSSGIFMKKEDSFHGTNQADDDIMC
LLDNFPLAVPVPEWYEDEDDKNNCNGQSSNVTNCDHIAENQAEDSKSPALTLNSGTRNHDW
EFGGCCWNNMPSFC
PhMYB101
MAPNGGGVKTSLARNISHGGTRHVLKKGPWTAAEDAILMEYVKKHGEGNWNAVQRNSGLM
RCGKSCRLRWANHLRPNLKKGAFSLEEERLIVELHAKLGNKWAHNEIKNYWNTRLKRRQRA
GLPIYPQDIQPQNQHEHNISSLFDHPQNSNFINPPLSLLDIFNPSTMKPSVNSQQYQFNNPSPFL
TNTNNNNNNHFKLFHDPRVSLSLTLASSIRNSQLSSLVAPVPKTFSQGLTTSMQVPPLQHNYPSFS
TNTRPYTAISTNPNGLILGMGIETTTCSRSTGNDYMKATSSSDADNYNVVDPGLSRGNSGLLED
LLEESQTLTRAEKIEENCLVDENEASQGKLVWEEYGLTEESTYSFAHGGDDTTPNSSSGITTKDA
SLELANQVDDDIMRFLDNFPVAVPAPDWCDDENEQNNTCNGHYFEPRDQMD
PhMYB102
MKTCAVRDSDEKDKGMGHHCCSKQKVKRGLWSPEEDEKLVRHITTHGHGCWSSVPKLAGLQ
RCGKSCRLRWINYLRPDLRRGSFTEQEERTIIDVHRILGNRWAQIAKHLPGRTDNEVKNFWNSC
IKKKLIAQGLDPNTHNLLSTHQNKNNKTNNSSKTKSYHQDSTSVFTIDTSTNKEVISMDIKATL
AALPPFLHSNNNTSSTYHYTTPIVPIIEYQNPSTFTLSERNNNGSTTTQHSVLDFASNTSINSTTN
NVSSSTLTPLEFGYINENCMWAGTGLEPTTLNPATGGTEEMQVQLQGEQFPIQTKFCDQEDVY
KVNQTVENTFDNSNFDFDFVDSALVPCGLYNSVNSMDQLSWDC
PhMYB103
MGHHSCCNQQKVKRGLWSPEEDEKLIRYITTHGYGCWSEVPEKAGLQRCGKSCRLRWINYLR
PDIRRGRFSPEEEKLIISLHGAVGNRWAHIASHLPGRTDNEIKNYWNSWIKKKLKKPSKPSTNTT
SCTEHQQQQRSQFSYNTTSQPEILFTQDLGVTKSQILQDSALFTSPNPLFYFDNGNSLETMTNN
VNDRSTNASLFQETSVLNSEFWQVDLQQQVHTSYTTGIHSNYLPPLLEMPHMEIPSSNNNMM
VEGQDHHQLNEWNMDTQQCCPSYLFWDQETGTLGGDHEFVDPNNTSNNIGQILSSFPSSL
PhMYB104
MGRIPCCEKENVKRGQWTPEEDHKLSSYIAQHGTRNWRLIPKHAGLQRCGKSCRLRWTNYLR
PDLKHGQFSEAEEQTIVTLHSVLGNRWSVIAAQLPGRTDNDVKNHWNTKLKKKLSGMGIDPV
THKPFSHLISEIATNLAPPQVPHLAEAALGCFKDEMLHLLTKKRIGFQFQQFGTSTAPSTSTVKV
EDNKEETIEKIKYGLSRAIKESDMLPSNKHWDPSGGARSTNLAEPSSGFPHVSDGGFQYNFASL
LHEDAAEGSPWNQSLCTGSTCTVGEQQQVHQLHKKLNSNDNCGEDNSDGAKETRNGSTTM
FHSDCILWDISSDDLLNPMV
PhMYB105
MRLLRFCVLASCDFQEMNFLTQNSLVMDYNKGRHCLSNGSTDGKVLENPWIFHSMVEDENG

TKLNVETEEVDLVGRKNNGGTKLCARGHWRPHEDAKLKELVAQYGPQNWNLIAEKLEGRSG
KSCRLRWFNQLDPRINRRAFSEEEEERLLTAHKMYGNKWAMIARLFPGRTDNAVKNHWHVI
MARKHREQNSVYRRRKPSNMQQFHSMGFPHVSGSDNAIQSNNLNSDSTISSTTNNMDEHCAS
TCTDLSLTPSSSKVIFMKRLTHMQHHHHPLLEASKGFSRDAQEVKMESGVHPKFLHGKEPRAD
TVDASHQYGCGGSDTNSDISASESVANNMTNVKMYGQNHENVKLVEKQMKSKTMPAFIDFL
GVGAT
PhMYB106
MTSYSCSPMGTTTTTTSMGMFYADMNSLSITSINYVTSDGGVGENSNETIDLNASCCFNEEKQIT
MHSNFSSGNNGKEIESGQSKLCARGHWRPAEDAKLKELVAIYGPQNWNLIADKLRWFNQLDP
RINRRAFTEEEEERLMAAHRLYGNKWAMIARLFPGRTDNAVKNHWHVIMARKYREQSSAYRR
RKMGQFVYRRTTTSLVEEEDSSFVSSNSSSGKEVVAATMKGENPTPIISGQTAANPFASLKINND
GPSGWVVYGPNGSSHMAASAGEAAPPSTNVIVGMK
PhMYB107
MARTRCYDKSGLKKGTWTPEEDRKLAAYVTRYGCWNWRQLPKFAGLARCGKSCRLRWLNYL
QPNIKRGNYTKEEDQIIMMLHAEIGNKWSAIAAHLPGRSDSEIKNHWHTSLKKRSTQEESTSST
NSKKRSPINSKYISSSRKKRSGSGNQISVKSNVPQLSPKQSCSEVSSCASIHVESMHVESEGYQEEY
LFEESSGISFWTEPFLVDNSSTTNDFFVPSELDHGLVSPFSPVISFDEFICSYD
PhMYB108
MGRTRCYDKSGLKKGTWTPEEDRKLTAYVTRYGSWNWRQLPKFAGLSRCGKSCRLRWLNYLQ
PNIRRGNYTKEEDEMIMKLHAEIGNKWSVIAAHLPGRSDNEVKNHWHTSLKKRSTQEESTSST
GSERRSNNSKYISSSRNKRRENGTQISANSNIPQLSPKQSCSELSSCASVDQHVENMHAEREVFQ
EEYLFEESSLISFWTEPFIVDNFSTTSDFFVTSQTDGGLVSPFSPFTRVIWVIFQFCAYHPWHLDGT
PDKRDISLAALVVSKSKSLVESFDTKLKENMMNDEPKKLIIELSVLKMKKLRNHCRPCARAVDP
RERSERNNGGRLEKRRAPVMNRQSNRWPPHA
PhMYB109
MEEAEQQSDFKLIDAAVNGGDTMAVDENNGGGGGGGKVKGPWSQDEDTVLSELVSKFGAR
NWSLIARGIPGRSGKSCRLRWCNQLDPSVKRKPFTEEEDNIIIQAHAVHGNRWASIAKLLPGRT
DNAIKNHWNSTLRRRFAGLKRVNPSHFEMLDSSNIDRVKATSEETRSGDDHNQSKSLEGREAS
VQQCKRTLLEDRAPVSEMFGVPEKVAHLVSGSCHSTINENNPSVSRPTAKVGAFDVHNLSSASF
ASSRTVPIQGNLLQAFSPDFGICKLLEGLPDEPIIPSSCGHGCCSAHSKSSSASSLLGPEFVEYEDLP
AVSSHELFAMATDLNNIAWIKSGLENAGKLPDKTTSGRDHQGSSTSTPMPMPMTSFVLPAQVE
SLS
PhMYB110
MVRAPCCEKVGIKRGRWTAEEDELLLKYIQANGEGSWRSLPKNAGLLRCGKSCRLRWTNYLR
PNLKRGKFTSEEDETIFKLQCSLGNRWSLMASYLPGRTDNEIKNYWNSHLRRRIYTFGMKKKPI
KTAAEMPNKTIVADGLNCESLKKRGRVSRSKAKKYNNNTTTTTTAYISTLKPKSSGVGAGGGAI
CSEGDSIVDAGIGLDIQQHDEDHAGSAIGKPRNEETEGTNQKHINATAEKQEVRNGILSFEEQG
QQVLDEHILIGPHEKNVGDETVHLQQPNYCLHDFGNQVSLSGVLEVDEESHENWWSTMNSD
NFLEDELWVDQCSSLDLEFGSIEECDDISLPLVAYSLVELLGPCVLVAIGKIWESVGCSPSGSRKFSS
LLRLGYVQGWAVKLMHCKRKDDDDGDGGDDDEDSSCISTVEFSFSTKEGFVKFRTNFCGCFG
GMFCSKVSIS
PhMYB111
MGRAPCCEKVGLKRGRWTAEEDETLTNYILTNGEGSWRSLPKNAGLLRCGKSCRLRWINYLRS
DLKRGNITSEEENIIIKLHATLGNRWSLIAGHLPGRTDNEIKNYWNSHLSRKVESLRIPSDEKLPQ
AVVDLARKGALKQIKRKVGRTSRSTMMRNRKSKNSAVSSLSMPKQLKESSEPLNATVPIMPSTP
NLEKEALSRTSTISSWLDGNNAMDSMQKEVANIAAPNPLMESREAHSSLSSDGMEWLEEIMPL
VIDDQDMDPNFIFTCLENGQGESAVKVTEEADNNFLNTSKINERDNNNRESSEAAVSINIHDE
QVHEKSNETSSLMKDEGTFDQWDWKEITHDGGKGWPWDDNNSLQLWDTTTDDTGFFQNCI

NEATVEMDSVHFENQKHSALVAWLLS
PhMYB112
MEGGSGGDGSAYQPYQNIPTNLSIFQQGPPLIAINRYLLSHQSQFSSQQSVNNQSIFSQCGGLCD
FSSYSGISHANGISWSSVPEPSFVDRIFLSHEQNGLHWNNQENGIAEEMISSQQNSKEAGKKAK
EGPSSTTVLVKGQWTEEEDRKLVKLVKQFGMRRWAQIAENMVGRAGKQCRERWHNHLRPDI
KKDAWSEQEELILVEAHQQIGNKWAEIAKKIPGRTENSIKNHWNATKRRQHSRRNKFKKQEK
DGQNDSKYRSNILRDYIRSKYFSDNSPPTASTPSNSISTNATPPYSDDDSPSLLTRQTYDEEMNFM
QNLFGENSLVENNGKVAEAMEAKTRQCPFDNKSSSSSCPFNSLVAGYTKSDNQIVNDNSEYGY
NMYMADHYSKVTQQSDSDQCSGIYLSYLLDHSTSANSLPCSGSIGYGNMNAGTLVNQGSSSSK
GNKEVDLMEIVSSTLYRQQTSHTTFFN
PhMYB113
MVGMMGWGANSDQQEWRKGPWTPEEDKLLSEYVNLHGEGRWSSVSRCAGLNRTGKSCRLR
WVNYLRPGLKRGHITPQEEGIIIELHALWGNKWSTIARYLPGRTDNEIKNYWRTHFKKKEKASS
KQDKRKTLRQRINNQPQLQHDTNYKFSPQPEEVIMQNSDEINEHNDTLTFTYHPNMENTIVDL
PPVRTSSDISYIWTDNFVMDGLWGGLWNLDVDDHSQPASDKCKVAIQNQPTDYQ
