# Supplementary material for: PhMYB37 Promotes Shoot Branching in Petunia
Source: Genes (Basel). 2022 Nov 8;13(11):2064. doi: 10.3390/genes13112064 (PMC9690887; doi:10.3390/genes13112064)
Supplement: Supplementary file 1 [file genes-13-02064-s001.zip › Table S2. Primer names and sequences used for polymerase chain reaction amplification.pdf]

Table S2. Primer names and sequences used for polymerase chain reaction amplifification.

Primer name	Primer sequences (5′-3′)
MYB37-qRT-F	GACTCTGCTGGGCTCAGTATT
MYB37-qRT-R	CATTAGCTCCTTTCTCAAACC
GAPDH-F	CAAGGCTGGAATTGCTTTGAG
GAPDH-R	CACCACTTTACTCCACTGATGCA
MYB37-SL-F	ATGGGAAGGACACCTTGTTGTGA
MYB37-SL-R	TCAGATAAAAGTGGATGGGAAAT
MYB37-vigs-F	CGGAATTCTCCATTATAGCATCTAAGCTACC
MYB37-vigs-R	CGGGATCCATTAATACTGAGCCCAGCAGAGT
Tublin-F	GAGCCTTACAACGCTACTCTGTCTGTC
Tublin-R	ACACCAGACATAGTAGCAGAAATCAAG
